# A novel combinatorial approach using sulforaphane- and withaferin A-rich extracts for prevention of estrogen receptor-negative breast cancer through epigenetic and gut microbial mechanisms

**DOI:** 10.1038/s41598-024-62084-1

**Published:** 2024-05-27

**Authors:** Mohammad Mijanur Rahman, Huixin Wu, Trygve O. Tollefsbol

**Affiliations:** 1 Department of Biology, University of Alabama at Birmingham, 902 14th Street South, Birmingham, AL 35294 USA; 2 Department of Microbiology, Heersink School of Medicine, University of Alabama at Birmingham, Birmingham, AL 35205 USA; 3 O′Neal Comprehensive Cancer Center, University of Alabama at Birmingham, 1824 6th Avenue South, Birmingham, AL 35294 USA; 4 Integrative Center for Aging Research, University of Alabama at Birmingham, 933 19th Street South, Birmingham, AL 35294 USA; 5 Nutrition Obesity Research Center, University of Alabama at Birmingham, 1675 University Blvd, Birmingham, AL 35294 USA; 6 Comprehensive Diabetes Center, University of Alabama at Birmingham, 1825 University Blvd, Birmingham, AL 35294 USA; 7 University of Alabama at Birmingham, 3100 East Science Hall, 902 14th Street South, Birmingham, AL USA

**Keywords:** Breast cancer, Cancer prevention, Cancer therapy, Cancer, Molecular biology, Epigenetics, Microbial communities, Microbiome

## Abstract

Estrogen receptor-negative [ER(−)] mammary cancer is the most aggressive type of breast cancer (BC) with higher rate of metastasis and recurrence. In recent years, dietary prevention of BC with epigenetically active phytochemicals has received increased attention due to its feasibility, effectiveness, and ease of implementation. In this regard, combinatorial phytochemical intervention enables more efficacious BC inhibition by simultaneously targeting multiple tumorigenic pathways. We, therefore, focused on investigation of the effect of sulforaphane (SFN)-rich broccoli sprouts (BSp) and withaferin A (WA)-rich Ashwagandha (Ash) combination on BC prevention in estrogen receptor-negative [ER(−)] mammary cancer using transgenic mice. Our results indicated that combinatorial BSp + Ash treatment significantly reduced tumor incidence and tumor growth (~ 75%) as well as delayed (~ 21%) tumor latency when compared to the control treatment and combinatorial BSp + Ash treatment was statistically more effective in suppressing BC compared to single BSp or Ash intervention. At the molecular level, the BSp and Ash combination upregulated tumor suppressors (p53, p57) along with apoptosis associated proteins (BAX, PUMA) and BAX:BCL-2 ratio. Furthermore, our result indicated an expressional decline of epigenetic machinery HDAC1 and DNMT3A in mammary tumor tissue because of combinatorial treatment. Interestingly, we have reported multiple synergistic interactions between BSp and Ash that have impacted both tumor phenotype and molecular expression due to combinatorial BSp and Ash treatment. Our RNA-seq analysis results also demonstrated a transcriptome-wide expressional reshuffling of genes associated with multiple cell-signaling pathways, transcription factor activity and epigenetic regulations due to combined BSp and Ash administration. In addition, we discovered an alteration of gut microbial composition change because of combinatorial treatment. Overall, combinatorial BSp and Ash supplementation can prevent ER(−) BC through enhanced tumor suppression, apoptosis induction and transcriptome-wide reshuffling of gene expression possibly influencing multiple cell signaling pathways, epigenetic regulation and reshaping gut microbiota.

## Introduction

Breast cancer (BC) is a global health burden for women's health. In the United States, BC was the second leading cause of cancer mortality with the highest incidence rate in women according to cancer statistics, 2023^1^. Among the types of BC, estrogen receptor-negative [ER(−)] triple negative/basal like (TNBC) BC is the most aggressive type, posing the highest threat to BC patients due to poor prognosis and a lack of targeted therapy^2^. Conventional therapeutic options against ER(−) BC often lead to undesirable short-term or long-term side effects, which has led to intensive investigation for safe and effective approaches for the prevention and treatment of BC^3,4^. Further, diet has been identified as a key factor explaining geographical and racial variations in BC incidence worldwide. Several epidemiological studies and meta-analyses also suggest a strong association of high fruit and vegetable consumption with an overall reduction in BC incidence^5,6^. Therefore, investigating dietary intervention for ER(−) BC at the molecular level constitutes an important scientific goal to explore novel and safe therapeutic as well as preventive strategies against BC.

Sulforaphane (SFN) is an isothiocyanate with potent histone deacetylase (HDAC) inhibitory activity and is enriched in cruciferous vegetables (e.g., broccoli sprouts, cabbage, cauliflower, kale). Previous studies from our laboratory demonstrated a profound anti-BC effect of SFN-rich broccoli sprouts (BSp) diet *in vivo*^7^. Interestingly, we have observed a stronger anti-BC effect in vivo and in vitro by combining SFN-rich BSp with other compounds with complementary epigenetic mechanism targeting ability^8,9^. It is worth mentioning that the anti-BC effect of a combinatorial BSp supplement was partially attributed to gut microbiota reshuffling, in addition to the direct targeting of epigenetic mechanisms^9^. On the other hand, withaferin A (WA) is a steroidal lactone abundant in Ashwagandha (*Withania somnifera*) and is known to be a DNA methyltransferases (DNMT) inhibitor^10,11^. WA exhibits potent anti-BC effect in both chemoprevention and therapeutic settings^12^. WA molecular targets have been well characterized including p53, FOXO3a, STAT3, ERα, ERK, JAK, IAP and NF-κB in BC^13^. Additionally, we have shown that SFN and WA combination targets multiple epigenetic pathways to induce cell cycle arrest and apoptosis in MCF-7 and MDA-MD-231 BC cells *in vitro*^14,15^.

Since breast tumorigenesis involves numerous combinations of genetic and epigenetic alterations, BC can be classified as an epigenetic disease. Increasing evidence suggests a global reshuffling of DNA methylation, histone acetylation, and histone methylation throughout the neoplastic transformation and progression of BC^7^. Several other studies have documented epigenetic abnormalities neutralizing capability of dietary phytochemicals to prevent BC. In this context, our laboratory has reported that interfering with epigenetic mechanisms including DNA methylation and histone deacetylation through dietary interventions can contribute to BC prevention possibly through the direct targeting of epigenetic mechanisms, alteration of regulatory non-coding RNA expression and shifting gut microbiome community in BC^16^. Notably, nutritional compounds may help to reshape gut microbial community while the gut microbiota plays a crucial role in synthesizing metabolites important for metabolic homeostasis^17,18^. For example, gut microbiota can ferment dietary fibers to produce short-chain fatty acids (SCFAs) such as butyrate, acetate, and propionate. Several of these SCFAs have been identified to alter epigenetic regulation and immune response in host cells^18^. However, past investigations from our laboratory, along with others, have successfully developed a novel strategy of combining two or more dietary phytochemicals to target BC^7^. Since the human food commonly consists of multiple nutritional ingredients, combinatorial dietary approach offers a practical and feasible therapeutic strategy for BC prevention by directly and/or indirectly targeting epigenetic mechanisms. Additionally, combinatorial approach offers advantage over single-agent therapeutic methods by effectively tackling tumor diversity, broadening the therapeutic window, and overcoming therapeutic resistance^8,19,20^. In this context, we investigated anti-BC effect of a novel combination of SFN-rich BSp and WA-rich Ashwagandha in this present study.

In this current investigation, we hypothesized that consumption of combinatorial SFN-rich BSp and WA-rich Ashwagandha (Ash) diets could effectively counteract BC development and progression in a spontaneous mammary cancer transgenic mice model. Therefore, we examined the impact of BSp and Ash administration, both individually and in combination, on tumor growth and development in a transgenic mouse model C3(1)-SV40 Tag (C3), with a comprehensive investigation into the molecular and epigenetic mechanisms associated with the anti-tumor effect. Additionally, we explored how the gut microbial community was influenced by the combinatorial dietary intervention both before and after tumor development. Our findings suggest that the combined administration of BSp and Ash was efficient in preventing ER(−) BC mammary tumors, partly due to a synergistic interaction between BSp and Ash. Combinatorial BSp and Ash treatment enhanced the expression of tumor suppressors associated with cell cycle regulation and apoptosis-associated proteins. Moreover, combined BSp and Ash administration altered the expression of several epigenetic machinery alongside reshuffling the gene expression profile in mammary tumors, and also altered the composition of gut microbiota.

## Results

### Early life BSp and/or Ash treatment prevents ER-negative breast tumor development in C3 mice

**Figure 1:**
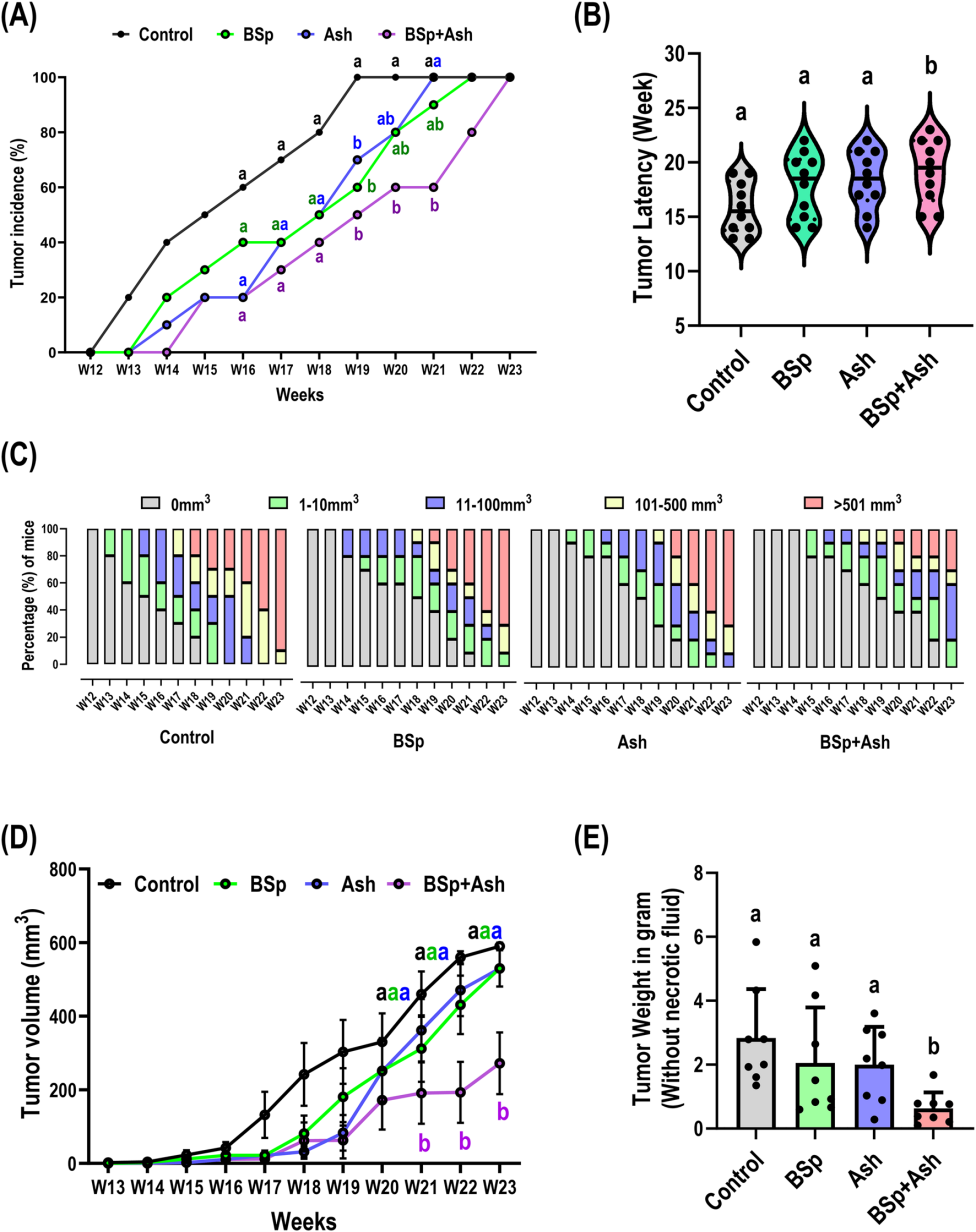
Early life BSp and/or Ash treatment prevents ER-negative breast tumor development in C3 mice. Effect of different single and combined administration of BSp and Ash on tumor incidence (**A**), tumor latency (**B**), tumor growth volume (**C**,**D**) and tumor weight measured at the termination point (**E**). Values are means ± SEMs. The significances of tumor incidence were analyzed using the chi-square test. Means that do not share a common superscript are significantly different at p ≤ 0.05. Significances of tumor volume among treatments were determined using two-way repeated measures ANOVA considering time and treatment as factors. Values are means ± SE, n = 10. Means that do not share a common superscript are significantly different at p ≤ 0.05. Tumor latency and tumor weight comparisons among the dietary groups were performed with One-way ANOVA analysis and Tukey’s HSD. Values are means ± SEM, n = 8–10. Means that do not share a common superscript are significantly different at p ≤ 0.05. Here, BSp: Broccoli sprouts; Ash: Ashwagandha; BSp + Ash: Broccoli sprouts and Ashwagandha combination.

In the current study, we used C3(1)-SV40 Tag (C3) transgenic mouse models that can spontaneously develop mammary tumors due to SV40 large T antigen (Tag) overexpression in epithelium of mammary grand in their early lifespan^21^. Functional inactivation of tumor suppressor p53 and retinoblastoma (Rb) by Tag lead to initiation of mammary hyperplasia around 8 weeks of age in hemizygous female mice in a hormone-independent manner that further develops to mammary intraepithelial neoplasia which resembles human ductal carcinoma in situ (DCIS) by around the 15th week of age^22,23^. Since the molecular and histological features exhibited in C3 mammary tumor model resembles TNBC, C3 mouse models have been successfully applied in many BC studies^22^. First, we were interested to evaluate the anti-tumorigenic effect of single and combined BSp and/or Ash treatment on breast tumor growth and development in C3 mice. We observed a delay in tumor development initiation in both the single compound treated groups (BSp: 13 weeks; Ash: 13 weeks) and combined BSp + Ash group (14 weeks) mice compared to the control group (12 weeks) mice (Fig. 1A,B). We also found an overall decrease in tumor incidence in BSp or Ash treated group as well as combined BSp + Ash treated group mice over time. Only combinatorial BSp + Ash treated mice exhibited a statistically significant reduction in tumor incidence from 19 to 21 weeks of age (Fig. 1A,B). In the case of tumor latency, a statistically significant increase in tumor latency (19.2 weeks) was observed only in combinatorial intervention group mice although single-compound interventions led to increased tumor latency (BSp: 17.9 weeks; Ash: 18.4 weeks) compared to control group mice (15.8 weeks) (Fig. 1C). The ratio of extended tumor latency for BSp, Ash and BSp + Ash group was 13.3%, 16.5% and 21.5% of control treatment respectively (Supplementary Table S1). Additionally, an additive interaction between BSp and Ash was predicted for extended tumor latency by combination treatment from combination index calculation (Supplementary Fig. S1). In the case of tumor volume, every single compound and combinatorial treatment led to a global reduction in tumor volume over time when compared to the control group. We also found a statistically significant decline in tumor volume only upon combinatorial treatment at 21 weeks of age and thereafter (Fig. 1D). Concordant with tumor volume reduction, combinatorial BSp + Ash administration resulted in a statistically significant reduction in tumor weight due to a synergistic interaction between BSp and Ash (Fig. 1E; Supplementary Fig. S1). The calculated tumor growth inhibition rate for BSp, Ash, and BSp + Ash group was respectively 27.5%, 29.4%, and 75.1% (Supplementary Table S1). Importantly, we observed no detrimental effects of BSp and/or Ash supplementation on mouse growth performance and hepatic function in C3 mice (Supplementary Fig. S2). In summary, combined BSp + Ash treatment was statistically more effective in suppressing tumor growth and development, as manifested by a delayed onset of tumors, lower tumor incidence, smaller tumor size, and lighter tumor weight in comparison to the outcomes seen in the BSp and Ash treatment groups. The anti-tumorigenic impact of the BSp + Ash combination can be attributed in part to the synergistic interaction between BSp and Ash.

### Dietary BSp and/or Ash treatment increases tumor suppressor expression in C3 mice

**Figure 2:**
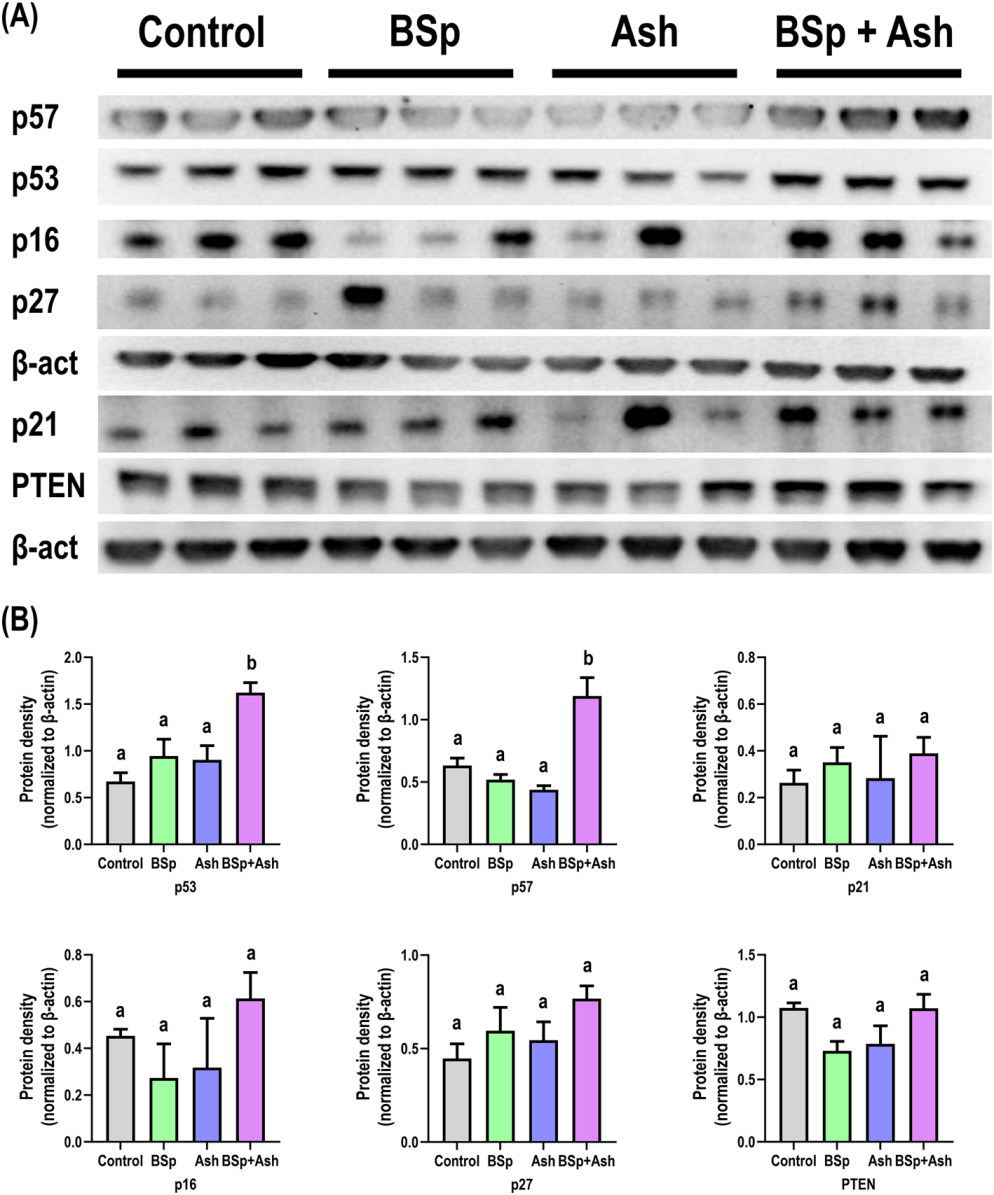
Expressional changes of key tumor suppressors proteins in the mammary tumor of single and combined BSp and Ash treated mice. The top panel shows the cropped images from an imaging system. The bottom panel shows the statistical analysis of band intensity calculated on the images using ImageJ. Graphs represent β-actin normalized protein expression level with each bar representing the mean ± SE (n = 3). Means that do not share a common superscript are significantly different at p ≤ 0.05 upon One-way ANOVA and Tukey’s post-hoc analysis. Here, BSp: Broccoli sprouts; Ash: Ashwagandha; BSp + Ash: Broccoli sprouts and Ashwagandha combination.

We were next interested in investigating the molecular mechanisms responsible for the cancer inhibiting effects of BSp + Ash combination. We first analyzed the protein expression level of several tumor suppressors in the mammary tumor tissue samples from each treatment group (Fig. 2). Transcription factor p53 can transactivate genes including p21 to induce cell cycle arrest and/or apoptosis in response to DNA damage^24^. Cip/Kip family members (i.e., p21, p27, and p57) control cell cycle by inhibiting various cyclin-dependent kinases (CDKs) in addition to their role in apoptosis induction^25^. p16 is an Ink4 family member that inhibits CDK4 and CDH6 in order to prevent Rb phosphorylation and subsequently trigger G1 arrest^26^. Tumor suppressor phosphatase and tensin homologue (PTEN) negatively regulate PI3K signaling for cell survival and proliferation besides regulating p53-dependent cell senescence^27^. According to our result, BSp alone treatment upregulated the expression of p53, p21 and p27 while Ash alone treatment enhanced p53 and p27 expression when compared to the control treatment (Fig. 2A,B). Feeding mice with combined BSp + Ash diet increased the expression of p53, p57, p21, p16 and p27. Furthermore, expressional upregulation of p53 and p57 in BSp + Ash-fed mice was statistically significant (Fig. 2B) compared to control as well as singly BSp or Ash treated mice. A synergistic drug-drug interaction was predicted between BSp and Ash on upregulating p57 and p53 expression in mammary tissue of combinatorial BSp + Ash treated mice (Supplementary Fig. S3). Together, combinatorial BSp + Ash treatment was more efficient in increasing tumor suppressor p53 and p57 in C3 mice than BSp or Ash treatment alone.

### Dietary BSp and/or Ash treatment enhances apoptosis in C3 mice

**Figure 3:**
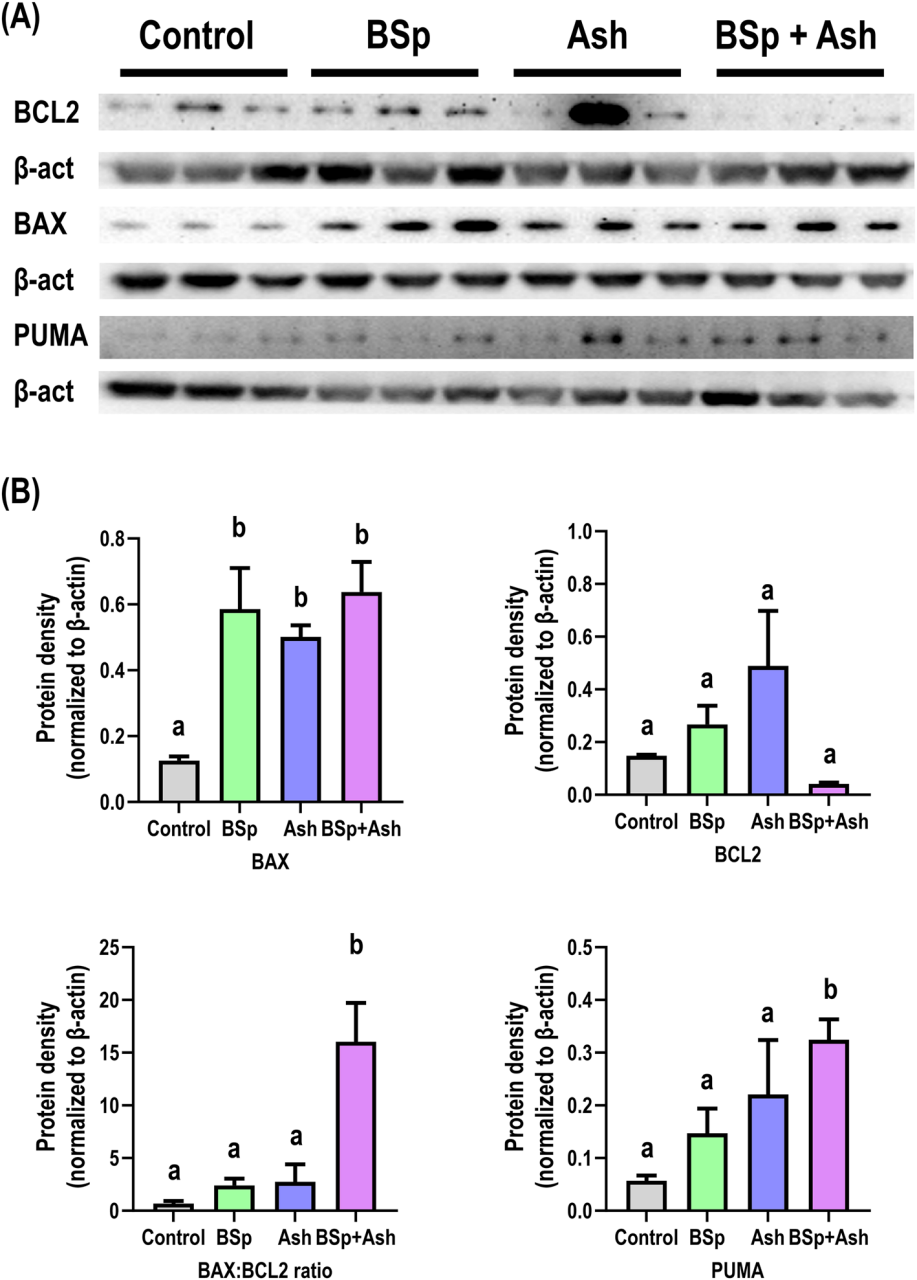
Expressional changes of key apoptosis-associated proteins in the mammary tumor of single and combined BSp and Ash treated mice. The top panel shows the cropped images from an imaging system. The bottom panel shows the statistical analysis of band intensity calculated on the images using ImageJ. Graphs represent β-actin normalized protein expression level with each bar representing the mean ± SE (n = 3). Means that do not share a common superscript are significantly different at p ≤ 0.05 upon One-way ANOVA and Tukey’s post-hoc analysis. Here, BSp: Broccoli sprouts; Ash: Ashwagandha; BSp + Ash: Broccoli sprouts and Ashwagandha combination.

We next measured the expression of apoptosis-associated proteins BAX, Bcl-2 and PUMA in mammary tumor tissue from each group in order to determine the impact of single and combined BSp and/or Ash administration on apoptosis induction in mammary tumor (Fig. 3). The results of western blot analysis suggested statistically significant upregulation of pro-apoptotic BAX protein expression as a result of singly administered BSp and Ash group mice compared to the control group mice. Singly administered BSp and Ash also increased the expression of PUMA and BAX:Bcl-2 ratio slightly without any statistically significant impact on anti-apoptotic Bcl-2 expression (Fig. 3A,B). In the case of combinatorial BSp + Ash-fed mice, we found a statistically significant upregulation of BAX, PUMA expression as well as BAX:Bcl-2 ratio (Fig. 3B). Additionally, we uncovered a synergistic interaction between BSp and Ash that led to a stronger uplifting of BAX:Bcl-2 ratio and PUMA expression in BSp + Ash fed mice compared to the effects observed in singly BSp and Ash administered mice (Supplementary Fig. S4). Taken together, BSp + Ash combinatorial treatment was more potent in enhancing apoptosis in C3 mice compared to single dietary interventions.

### Dietary BSp and/or Ash treatment alters the expression of epigenetic machinery in C3 mice

**Figure 4:**
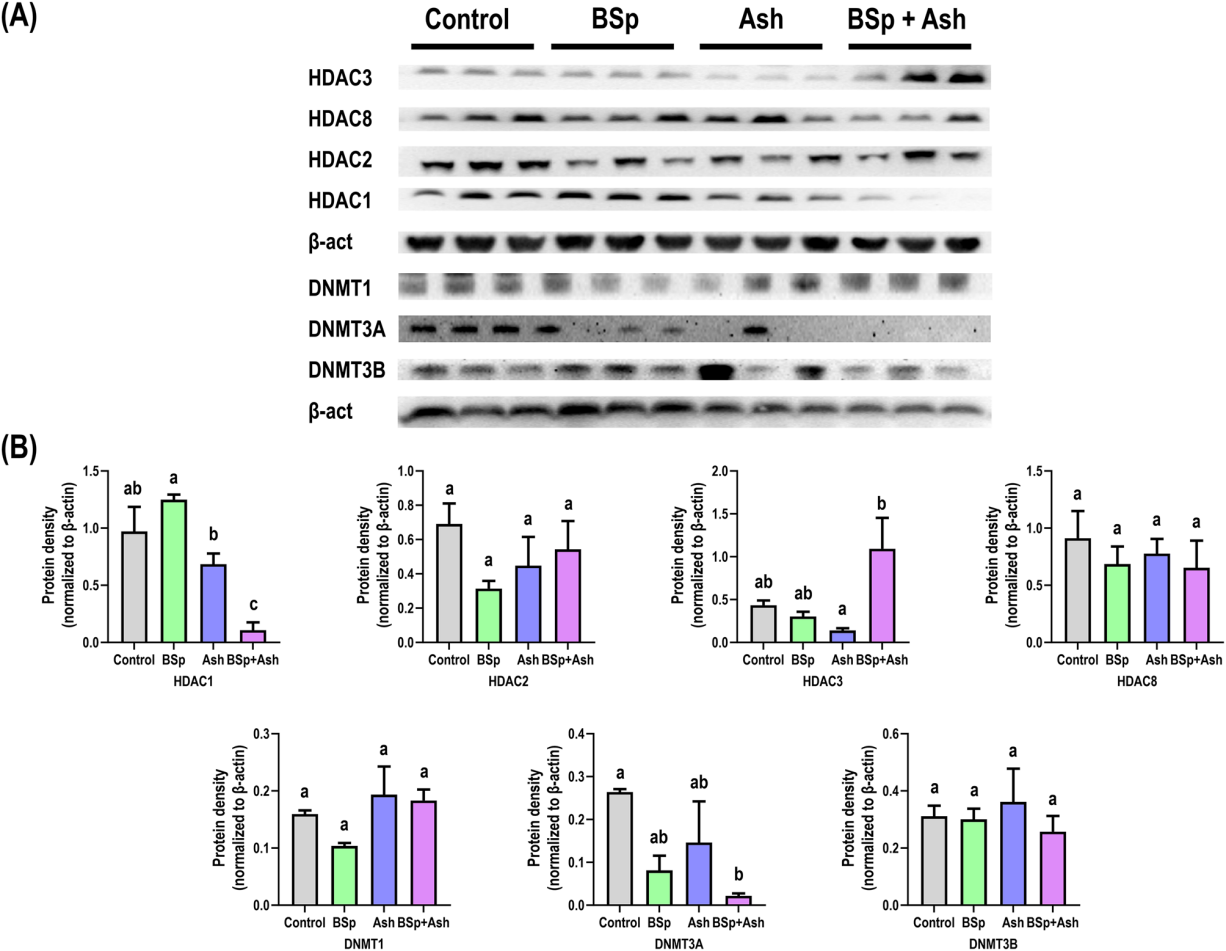
Expressional changes of key epigenetics modification-related proteins in the mammary tumor of single and combined BSp and Ash treated mice. The top panel shows the cropped images from an imaging system. The bottom panel shows the statistical analysis of band intensity calculated on the images using ImageJ. Graphs represent β-actin normalized protein expression level with each bar representing the mean ± SE (n = 3). Means that do not share a common superscript are significantly different at p ≤ 0.05 upon One-way ANOVA and Tukey’s post-hoc analysis. Here, BSp: Broccoli sprouts; Ash: Ashwagandha; BSp + Ash: Broccoli sprouts and Ashwagandha combination.

Earlier, overexpression of class-1 HDAC isoenzymes (i.e., HDAC1, HDAC2, HDAC3, HDAC4) have been reported in BC^28^. Additionally, DNMTs exhibited stage-specific overexpression, with maintenance methyltransferase DNMT1 overexpression observed in the metastatic BC and de novo methyltransferases (i.e., DNMT3A, DNMT3B) overexpression detected in the primary stage of BC^29^. Since SFN in BSp and WA in Ash are respectively known for their HDAC and DNMT inhibitory effect, we analyzed the expression class I HDACs, de novo methyltransferases and maintenance methyltransferase on the tumor tissue from all treatment groups (Fig. 4). As demonstrated by the result, both single compound interventions resulted in insignificant reduction of several class I HDACs including HDAC2, HDAC3, HDAC8 for BSp treatment and HDAC1, HDAC2, HDAC8 for Ash treatment (Fig. 4A,B). Notably, combinatorial BSp and Ash administration resulted in a significant reduction of HDAC1. On the other hand, DNMT1 and DNMT3A were slightly decreased in BSp-fed mice while Ash treatment reduced the expression of DNMT3A to some extent. However, combinatorial BSp and Ash administration significantly reduced the expression of DNMT3A (Fig. 4B). We also computed synergistic interaction between the BSp and Ash for HDAC1 and DNMT3A downregulation by combined diet that resulted in a robust effect compared to the BSp and Ash treatment alone (Supplementary Fig. S5).

### Dietary BSp and/or Ash treatment induce alteration of transcriptome in mammary tumor of C3 mice

**Figure 5:**
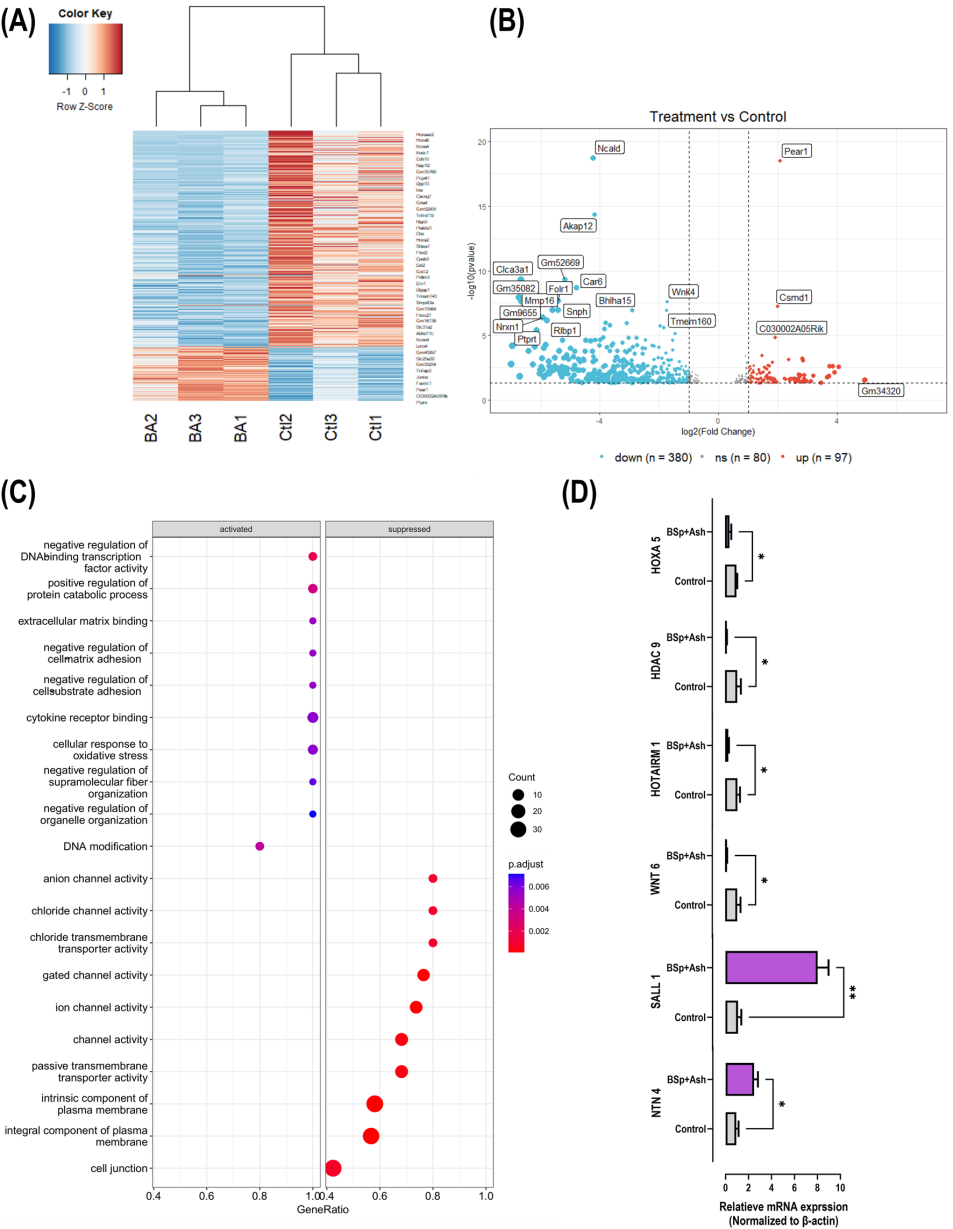
Dietary BSp and/or Ash treatment induce alteration of transcriptome in mammary tumor of C3 mice. (**A**) Heatmap representing differentially expressed mRNAs in C3 mice due to combinatorial BSp and Ash treatment. Each row represents differentially expressed mRNA, and each column represents biological replicates. Here, Ctl indicates control diet treated control group samples (n = 3) while BA indicated combinatorial BSp + Ash treated test group samples (n = 3). Blue color denotes downregulation and red color denotes upregulation. (**B**) The volcano plot shows distribution of DEGs in C3 mice as a result combinatorial BSp + Ash treatment. Here, gray dots indicate mRNAs that were not significantly expressed, blue dots represent downregulated mRNAs and red dots indicate upregulated mRNAs [Benjamini–Hochberg FDR < 0.05 and |log2(fold-change) |> 1). (**C**) The dot plot representing the gene ontology enrichment terms for the differentially expressed mRNAs in C3 mice. The dot size represents the number of enriched genes while the dot color represents activated (blue) and suppressed (red) genes. (**D**) Validation of differentially expressed mRNA with qRT-PCR. Graphs represent β-actin-normalized expression level of corresponding mRNA with each bar representing the mean ± standard error (SE) of three biological replicates (n = 3). The asterisks indicate statistical significance (* p ≤ 0.05, ** p ≤ 0.01, *** p ≤ 0.001, **** p ≤ 0.0001) upon unpaired T-test. Here, BSp + Ash indicates broccoli sprouts and Ashwagandha combination treatment.

Since dietary BSp and/or Ash treatment altered the expression of a several key molecular players associated with transcriptional regulation, apoptosis, and epigenetic regulation, we further aimed at investigating the impact of combinatorial BSp and Ash on the transcriptome changes by RNA sequencing analysis. We started with total RNA extraction from mammary tissue harvested at the termination point of the study and subsequently prepared RNA-seq pair-end library. Raw mRNA-seq data was first processed for quality check and eventually used for differential gene expression analysis. From the unsupervised principal component analysis (PCA), we observed a clear separation of control group samples from combinatorial group samples along the first component (73%). Control group samples further clustered separately in different clades from the combinatorial group samples in hierarchical cluster dendrogram (Supplementary Fig. S6A,B). Next, we performed differential gene expression analysis with DESeq2 package utilizing raw count matrix obtained after read alignment and counting. Our inspection on model fitness suggested a good fitting of DESeq2 model to our current data set (Supplementary Fig. S6C). We selected a 5% false discovery rate (FDR < 0.05) and a fold-change (|log2(FoldChange)|> 1) cutoff greater than 1 to determine the significance of a differentially expressed (DE) mRNA. Overall, we identified 477 differentially expressed mRNA in mammary tumor of combinatorial BSp + Ash treated mice compared to control group mice. Out of these differentially expressed mRNAs, 97 mRNAs were upregulated, and 380 mRNAs were downregulated. We plotted all differentially expressed mRNAs due to combinatorial treatment in a heatmap to better visualize overall mRNA expressional changes (Fig. 5A) across control and combinatorial treatment group. A comprehensive list of DE mRNAs is available in Supplementary Table S2. Generation of volcano plot besides heatmap further enhanced our understating on the expression level changes of DE mRNAs (Fig. 5B). Finally, we performed gene ontology (GO) analysis to ascribe biological theme to DE mRNAs and identified significantly affected themes (adj p < 0.05) in the categories of biological processes, molecular function, and cellular components. Interestingly, GO analysis revealed that activated DE mRNAs category were associated with DNA modification, transcription factor activity and negative regulation of cell adhesion while suppressed DE mRNAs category were enriched with cell junction component, integral plasma membrane component (Fig. 5C). In the functional category, DE mRNAs were associated with channel activity, passive transmembrane transporter, gated channel activity, structural constituent of synapse, phosphoric ester hydrolase activity, metal ion transmembrane transporter, calcium-dependent phospholipid binding and cyclase activity (Supplementary Fig. S6D). Pathway enrichment analysis further indicated that combinatorial BSp and Ash treatment could impact multiple signaling pathways including Hipo-, chemical carcinogenesis-, TNF- and estrogen-signaling pathway influencing multiple molecular components (Supplementary Fig. S6E,F). We finally evaluated the expression of several candidate genes by qRT-PCR (Fig. 5D) for validation of our genome-wide analysis including multiple candidates from the upregulated and downregulated gene lists. Consistent with our genome-wide analysis, we found significant upregulations of *SALL1 and NTN4* and downregulation of *HOXA 6, HDAC 9, HOTAIRM1* and *WNT 6* in C3 mice because of combinatorial BSp and Ash treatment. In this regard, *SALL1* acts as a tumor suppressor by recruiting nucleosome remodeling and deacetylase complex to trigger tumor cell senescence in BC^30^. The expression of *NTN4* is downregulated in BC, whereas NTN4 overexpression inhibits migration and invasion of MDA-MB-231 cells^31^. Conversely, the oncogene *HOXA 6* has been reported to aggravate cancer metastasis^32,33^. Overexpression of Class II a HDAC 9 promotes cell proliferation and therapeutic resistance against HDAC inhibitors in BC^34^. Downregulation of long non-coding RNA HOTAIRM1 promotes tamoxifen resistance in ER + BC cells^35^. WNT6 is overexpressed in BC and promote cell proliferation and migration^36^.

### Early life BSp and/or Ash treatment alters gut microbial composition before and after the tumor development

**Figure 6:**
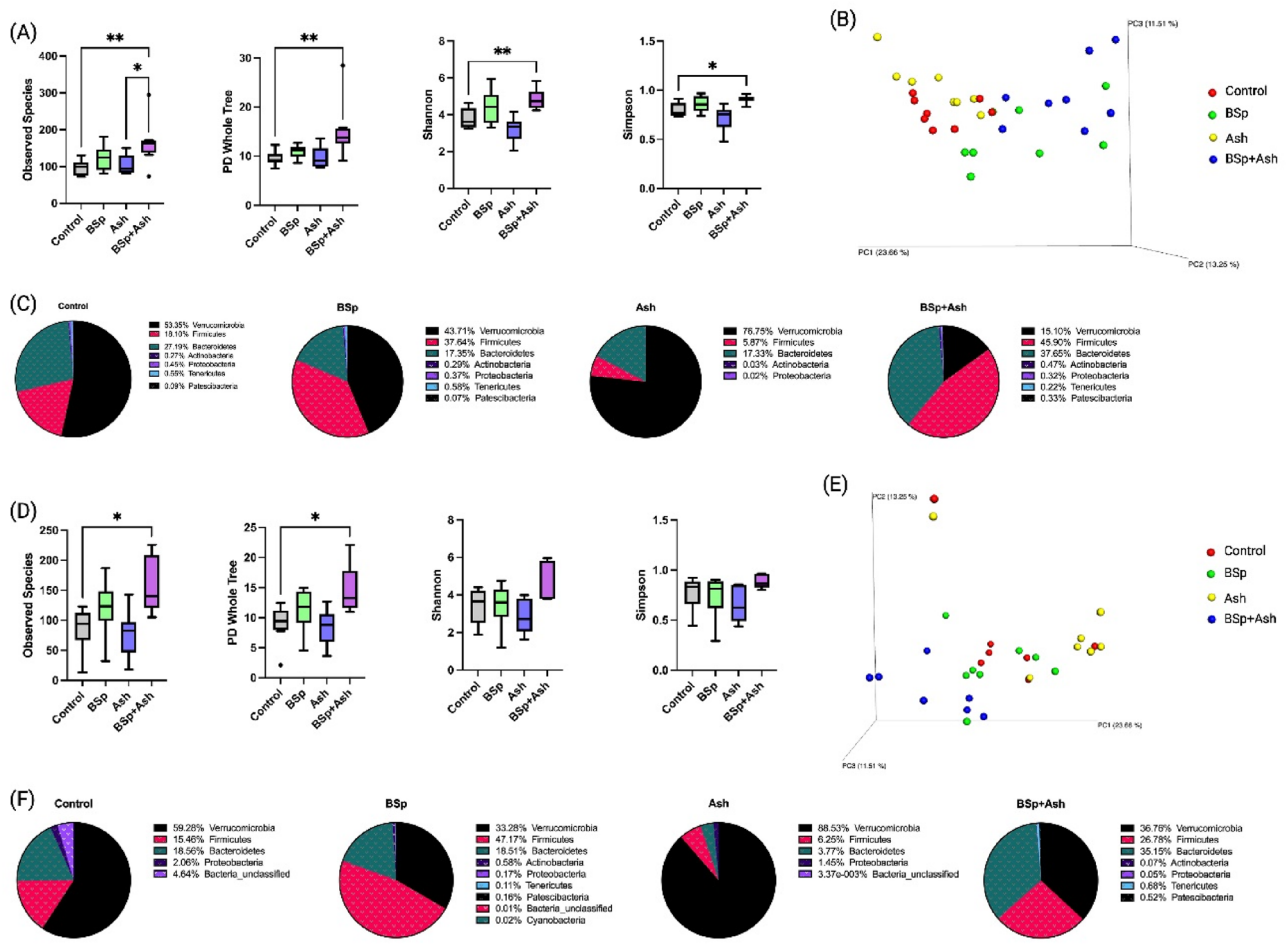
Gut microbial composition changes in dietary treatment groups before and after tumor onset. (**A**) Alpha-diversity before tumor onset: observed species, PD whole tree, Shannon and Simpson diversity. The asterisk indicates statistical significance (*p ≤ 0.05, **p ≤ 0.01) upon One-way ANOVA and Tukey’s post-hoc analysis. Here, BSp: Broccoli sprouts; Ash: Ashwagandha; BSp + Ash: Broccoli sprouts and Ashwagandha combination. (**B**) Bray Curtis 3D-PCoA plot before tumor onset. The red dots indicate control group samples, green dots indicate BSp group samples, yellow dots indicate Ash group samples, blue dots indicate combinatorial BSp + Ash group samples. Statistical analysis of Bray–Curtis and weighed Unifrac tests of beta diversity was performed using permutational multivariate analysis of variance (PERMANOVA). Statistical significance: control-BSp (p = 0.007), control-Ash (p = 0.043), control-combination (p = 0.003); and with weighed Unifrac control-BSp (p = 0.462), control-Ash (p = 0.042), control-combination (p = 0.002). (**C**) Phylum level changes of microbial community in dietary treated groups before tumor onset. The top 10 abundant phyla in respective groups are represented in pie charts. **D**) Alpha-diversity after tumor onset: observed species, PD whole tree, Shannon and Simpson diversity. The asterisk indicates statistical significance (*p ≤ 0.05) upon One-way ANOVA and Tukey’s post-hoc analysis. Here, BSp: Broccoli sprouts; Ash: Ashwagandha; BSp + Ash: Broccoli sprouts and Ashwagandha combination. (**E**) Bray Curtis 3D-PCoA plot after tumor onset. The red dots indicate control group samples, green dots indicate BSp group samples, yellow dots indicate Ash group samples, blue dots indicate combinatorial BSp + Ash group samples. Statistical analysis of Bray–Curtis and weighed Unifrac tests of beta diversity was performed using permutational multivariate analysis of variance (PERMANOVA). Statistical significance: control-BSp (p = 0.007), control-Ash (p = 0.043), control-combination (p = 0.003); and with weighed Unifrac control-BSp (p = 0.462), control-Ash (p = 0.042), control-combination (p = 0.002). (**F**) Phylum level changes of microbial community in dietary treated groups after tumor onset. The top 10 abundant phyla in respective groups are represented in pie charts.

Dietary supplements were digested and utilized by gut microbial species before their by-products and metabolites are transported to other parts of the body^9^. Previous research indicated an association between dietary phytochemicals and SCFA-producing gut microbiota^37^. Here, we also investigated the changes in gut microbial composition of C3 mice due to single or combinatorial dietary supplements. Fecal samples were collected before and after the onset of tumor, since tumor development has been found to have an impact on gut microbiota. According to the results of gut microbiota analysis before tumor onset, combinatorial treatment significantly increased alpha diversity compared to the control group (Fig. 6A). The Bray Curtis beta diversity analysis, as shown in the 3D PCA plot, demonstrated that individual samples in each group were clustered distinctly against control group samples which were statistically significant (Fig. 6B). Both BSp and combination groups had higher relative abundance of Firmicutes and lower relative abundance of Bacteroidetes and Verrucomicrobia. Ash group had substantially higher relative abundance of Verrucomicrobia and lower abundance of Firmicutes and Bacteroidetes (Fig. 6C). These results suggest that the combinatorial group had higher microbial diversity and distinct microbial composition before the tumor onset. Additionally, numerous significantly different bacterial taxa were identified compared to the control group, BSp (n = 12, at species level), Ash (n = 3, at family level), and combinatorial group (n = 14, at species level). BSp group had significantly higher relative abundance of *Ruminococcus*, *Muribaculaceae*, and *Lachnospiraceae xylanophilum group* (Supplementary Table S7). The Ash group had lower relative abundance of *Rikenellaceae* (Supplementary Table 8). The combinatorial group had significantly higher relative abundance of *Lachnospiraceae bacterium COE1*, *Ruminococcus*, *Coprostanoligenes group*, *Lachnospiraceae xylanophilum group*, and *Muribaculaceae* (Supplementary Table S9/Table S6). Overall, differences of bacterial composition were found in all dietary treatment groups among which the combinatorial group had more significant abundance of bacterial genus or species than single treatment groups when compared to the control group.

Gut microbial composition analysis after tumor development suggested that combinatorial treatment group also had significantly higher alpha diversity compared to the control group (Fig. 6D). We also observed differential clustering of individual samples against control group in the Bray Curtis beta diversity analysis (Fig. 6E) with statistical significance. We observed a higher relative abundance of Proteobacteria and Verrucomicrobia in control and Ash group respectively. BSp group mice had higher Firmicutes to Bacteroidetes ratio compared to control. The combinatorial group had relative abundance of both Firmicutes and Bacteroidetes, but lower abundance of Verrucomicrobia (Fig. 6F). The Kruskal–Wallis test suggested no significant difference of bacterial community was found in BSp and Ash groups compared to control (Supplementary Table S10-11). The combinatorial group had significantly abundant bacterial taxa (n = 3, at class level). Coriobacteriia, Bacterioidia, and Saccharimonadia decreased compared to the control group (Supplementary Table S12). Collectively, these data indicate that the combinatorial dietary treatment may have had greater impact on gut microbiota during mammary tumor progression. Additionally, longitudinal studies suggested that when comparing the gut microbiota composition between two different time points within each treatment group and the control group, no significant differences of relative abundant genus or species were found in control or treatment groups (n = 0, at genus or species level) (Supplementary Table S13-16). In conclusion, the gut microbiota composition within each treatment group was constantly shaped by BSp, Ash, and combinatorial dietary interventions, among which the combinatorial intervention had the most promising effect of BC inhibition in the current transgenic mouse model.

## Discussion

Many studies on breast cancer prevention have focused on individual compound interventions, despite the fact that the human diet typically includes a variety of nutritional components. Additionally, the effectiveness of single dietary interventions in BC management might be compromised by the risks of toxicity, incomplete therapeutic range, narrow targeting, and therapeutic resistance^8,19,20^. On the other hand, recent research suggests that a combinatorial therapeutic approach can yield promising efficacy in breast cancer chemoprevention due to potential anticancer properties and differential molecular targeting^7,38,39^. In this regard, we have reported that combination of SFN and WA can induce cell cycle arrest and apoptosis in MCF-7 and MDA-MD-231 BC cells in vitro. We also found that SFN and WA combination targets multiple epigenetic pathways including DNA methylation, histone deacetylation to exert their anti-BC effect^14,15^. Therefore, we were interested in examining if the in vitro findings with WA and SFN combination would be translatable to an in vivo scenario through a comprehensive investigation of molecular and epigenetic mechanisms.

We started our investigation by examining the impact of combinatorial broccoli sprout (BSp), a well-known source of SFN, and WA-rich Ashwagandha extract (Ash) diets on the latency, incidence, and progression of triple-negative BC. We selected C3(1)-SV40 Tag (C3) transgenic mouse model in the current investigation since C3 transgenic mouse is a widely used mouse model that develops mammary neoplasia spontaneously in female mice in a hormone-independent manner. The gene expression profile and histological features of mammary neoplasia in C3 mouse model resembles human basal-like triple-negative aggressive cancers^22,23^. Additionally, our lab has demonstrated dysregulation of multiple epigenetic pathways in C3 model which makes this mouse model suitable for studying both anti-BC and epigenetic mechanisms at the molecular level^8,40^. With respect to dose selection, we opted for 26% BSp because the safety and efficacy of 26% BSp diet, especially in a combinatorial scenario, has been proven in several investigations in our laboratory^8,9,41^. Likewise, we selected 0.16% Ash considering the in vitro dose safe evaluation and reported pharmacological safety evaluation data^42,43^. Furthermore, this dietary combinatorial regimen is physiologically attainable by consuming ∼ 234 g of BSp and ∼185 mg of Ashwagandha extract powder (6% WA) by an adult per day, respectively^44–46^.

Our phenotypic results from breast tumor development demonstrated a significant decrease in tumor incidence and extended tumor latency due to combined BSp and Ash administration. Additionally, we observed a noteworthy inhibition of tumor growth, as evidenced by smaller tumor volume and lower tumor weight resulting from BSp + Ash treatment. Our findings highlight that the combination of BSp and Ash was more effective in suppressing tumor growth and development, even though individually administered BSp and Ash exhibited anti-breast cancer effects to some extent. Overall, the tumor phenotypic data indicate that the combinatorial treatment has the ability to interfere with tumor growth kinetics in C3 mice. Since delayed tumor growth kinetics has been linked to prolonged tumor doubling times and surface shrinkage of the tumor mass at the growing edge, we propose that dietary BSp + Ash treatment may interfere with tumor growth kinetics by altering cell cycle regulation as well as inducing apoptosis.

We then focused on investigating the impact of combinatorial BSp and Ash treatment on the expression of several cell cycle associated tumor suppressors and apoptosis-associated proteins in mammary tumor tissue. Our results suggested a significant upregulation of p53 and p57 upon combined BSp and Ash treatment only. Both p53 and the Cip/Kip family member p57 have been reported to trigger cell cycle arrest at multiple stages, including G1 and G2/M phases in breast cancer^47,48^. We also found that expressional upregulation of p53 was concomitant with transcriptional down-regulation of *CCND1* and *CDK4* in BC cells due to combined SFN and WA treatment *in vitro*^13^. Additionally, p53 has also been implicated in apoptosis induction through the transactivation of Bcl2 family proapoptotic genes, including BAX, BID and PUMA. In line with this, we observed a significant upregulation of pro-apoptotic BAX, PUMA, and the BAX:Bcl-2 ratio due to combinatorial treatment. Indeed, the balance between Bcl-2 and Bax expression acts as a regulator for cell survival in response to apoptotic stimuli by altering the apoptotic threshold for cancer cells^49^. PUMA can further potentiate apoptotic threshold by inhibiting Bcl2 family antiapoptotic proteins^50^. Combinatorial treatment-induced BAX and PUMA upregulation; therefore, mechanistically decreased cellular resistance to apoptotic stimuli by lowering the apoptotic threshold and consequential increase apoptotic cell death in mammary tumor.

Earlier we reported the HDAC and DNMT inhibitory activity of SFN and WA in BC cells^14^. We took the findings into account to further study the impact of BSp and Ash diet on class I HDACs and DNMTs expression in mammary tissue. As anticipated, the combined BSp + Ash diet significantly declined the expression of HDAC1 and DNMT3A in mammary tumors. Importantly, SFN-rich in BSp has been proposed to trigger the kinase signaling pathway for cytoplasmic translocation and subsequent ubiquitination and/or Pin1-directed HDAC degradation^51^. On the other hand, WA available in Ash may impact the expression of multiple chromatin-modifying enzymes that may lead to expressional downregulation of DNMT^52^. Our previous in vitro study also demonstrated that down-regulation of HDAC1 and DNMT3A activity was associated with expression changes of p53, decreased Rb phosphorylation and elevated BAX:Bcl-2 ratio in BC cells^15^. Indeed, HDACs and DNMTs both play critical roles in regulating cell cycle progression and apoptosis as epigenetic regulators. Mechanistically, HDAC inhibition can interfere with cell cycle progression by abolishing Rb phosphorylation besides influencing the expression of cell cycle modulators, including p53, E2F, p21, and p27^53^. The expressions of p21 and p27 have been reported to be dysregulated by DNA methylation and histone deacetylation in multiple hematologic and solid cancers, including BC^54,55^. Furthermore, apoptosis regulator BAX and BCL-2 could undergo hypermethylation-mediated silencing in neoplastic situations^56^. Overall, our current results together with previous findings indicated that combinatorial BSp and Ash may influence epigenetic regulation to exert its anti-BC effect in ER(−) BC.

Our differential gene expression analysis revealed a global reshuffling of transcriptome due to BSp + Ash treatment. According to GO analysis, differentially expressed genes were associated with DNA modification, transcription factor activity, cell adhesion, cell junction and other processes. We further demonstrated transcriptional downregulation of *HDAC9* and *Hotairm1* by BSp + Ash treatment. HDAC9 is associated with enhanced cell proliferation and invasiveness of breast cancer^57,58^. On the other hand, *Hotairm1* is a long non-coding RNA that has been reported to promote tamoxifen resistance in breast cancer by enhancing HOXA1 upregulation. Mechanistically, HOTAIRM1 halts PRC2 complex mediated H3K27me3 deposition on the HOXA1 promoter by direct interaction with epigenetic modifier EZH2^35^. These findings, together with expressional down-regulation of HDAC1 and DNMT3A due to combinatorial treatment, reinforce our assumption that BSp + Ash-induced expressional change in epigenetic molecules may be linked to the observed alteration in the transcriptome.

We have identified several signaling pathways from our pathway enrichment analysis including Hippo-signaling, TNF-signaling, chemical carcinogenesis-signaling and estrogen signaling that are influenced by the combinatorial treatment mediated transcriptional alteration. Several of these influenced pathways have significant impact on the expression of genes associated with cell proliferation, cell cycle, cell adhesion, cell migration, cell invasion, cell survival, apoptosis, transcription factors, cell adhesion molecule, cell membrane components and leukocyte recruitment. This finding aligns closely with our experimental findings where we have demonstrated expression alteration of multiple genes associated with cell cycle and apoptosis. Additionally, pathway analysis enabled us to extrapolate on the molecular mechanisms that might be orchestrated by the combinatorial to BSp + Ash treatment to exert its anti-BC effect. Overall, further investigation could shed light on the signaling pathways that may be involved with the observed anti-BC effect of combinatorial BSp + Ash treatment.

Earlier it was reported that the BC patients have different breast and gut microbial composition compared to healthy controls^59,60^. Gut microbiota plays an important role in releasing metabolites such as SCFAs that are known to modulate epigenetic regulations and immune responses. Here, the gene expression analysis revealed that HDACs, WNT, and HOTAIRM1-HOXA1 were significantly decreased by BSp and Ash combinatorial treatment. Previous findings suggested that SCFA inhibited the expression of HDACs and WNT signaling. Therefore, we investigated potential association of gut microbiota including SCFA-producers that were shaped by the dietary supplements with tumor prevention and latency^61,62^. Technically, our study compared gut microbial community between treatment groups and the control group at two time points, before and after the tumor onset. Increased alpha diversity and beta-diversity were observed in combinatorial treatment group both before and after the tumor onset when compared to the respective control. The results indicated that combinatorial BSp and Ash treatment induced prominent changes of gut microbial composition in our ER(−) BC mouse model. Longitudinal studies further confirmed that dietary supplementation had constant impact on gut microbial composition over time, as the changes of relative abundance of analyzed species are not significantly different when compared longitudinally. Together, these results suggested that the combinatorial BSp and Ash treatment constantly shaped gut microbial composition toward health-benefiting bacterial communities before the tumor onset and during cancer progression.

Our results further indicated several possible mechanisms behind such health-promoting microbiota shift that may play a crucial role in observed anti-BC effect of combinatorial treatment. For example, combinatorial BSp and Ash treatment may shape gut homeostasis by maintaining Firmicutes to Bacteroidetes (F/B) ratio. We have observed an elevated Firmicutes to Bacteroidetes (F/B) ratio before and during BC progression due to BSp and combinatorial treatment whereas lower F/B ratio has been linked to higher risk of breast cancer^63^. However, such health promoting implication of the F/B ratio in inhibiting ER(−) BC needs further investigation. Several organisms that we identified in the combinatorial BSp and Ash treated mice are known as primary contributors of anti-inflammatory metabolites including pro-inflammatory *Eubacterium xylanophilum* and *Muribaculaceae*. For example, *Muribaculaceae* has been reported to decrease pro-inflammatory activity in the host intestine by reducing TNF-α and IL-6 and increase IL-10^64^. *Eubacterium xylanophilum* group was found to have a strong positive correlation with levels of proinflammatory cytokines^65^. Though we did not conduct any formal investigation on the impact of pro-inflammatory bacteria on observed anti-BC effect, we have identified TNF-signaling as one of the pathways impacted by combinatorial treatment. Thus, further investigation may be warranted to rule out how these pro-inflammatory bacteria contribute to observed anti-BC effect of combinatorial diet. On the other hand, *Ruminococcaceae *and *Muribaculaceae* have been recognized as producers of SCFAs^66,67^. Furthermore, a positive correlation has been reported between *Ruminococcaceae* abundance and butyrate/SCFA ratio *in vivo*^68^. Interestingly, several recent studies from our laboratory have reported that the gut microbiota can produce SCFAs to impede tumor progression. In this regard, we have reported that combinatorial BSp and green tea polyphenols diet could differentially affect the gut microbial composition with a concomitant rise of serum level of SCFAs including propionate and isobutyrate^18^. In another study, we have reported alteration of gut microbiota due to insulin supplementation with a simultaneous rise of serum propionate level. Furthermore, propionate was able to inhibit cell proliferation as well as HDAC and DNMT enzymes activity in multiple BC cells *in vitro*^69^. These findings encourage further study to uncover if microbial induction of SCFAs and consequential alteration of epigenetic regulation has any connection to observed anti-BC effect of combinatorial BSp and Ash treatment.

Throughout the investigation, we have observed synergistic interaction between SFN-rich BSp and WA-rich Ash both at the phenotypic (i.e., tumor inhibition) and molecular level which indicates that combined effect of BSp and Ash is more effective than either compound alone. There are multiple factors that may contribute to the observed synergistic interaction between BSp and Ash. Firstly, we designed our combination to target alternative epigenetic pathways. BSp is a well-known source of SFN that functions as a HDAC inhibitor whereas Ash is a rich source of WA which is a pronounced DNMT inhibitor. Crosstalk between epigenetic regulation pathways may be another possible mechanism for synergistic interaction of BSp and Ash combination which could be most impactful in our experimental context. Increasing evidence suggests that DNA methylation could influence the chromatin orientation by influencing histone modifications and vice versa^70^. DNMTs have been documented to engage in physical interactions with HDACs, and they can be jointly enlisted into transcriptional repressor complexes, such as MeCP2, to facilitate transcriptional repression^71^. DNMT and HDAC cooperativity may lead to their concerted efforts in transcriptional regulation. For instance, DNMTs may establish DNA methylation patterns that attract HDACs to further modify histones, creating a repressive chromatin environment with an enhanced suppression of gene activity^72^. Both BSp and Ash are a rich source of antioxidants that neutralize oxidative stress. BSp has been reported to induce Keap1-Nrf2 pathway whereas Ash administration increased levels of multiple antioxidant enzymes including superoxide dismutase and catalase^73,74^. Variance in antioxidative defense attained by BSp and Ash administration could lead to a comprehensive protection against oxidative damage and enhanced chemoprevention. Finally, BSp administration has been reported to inhibit NLRP3, IL-1β, IFN-γ, IL-17 and IL-23 IL-12 whereas Ash administration inhibits TNF-α, IL-1β, IL-6, IL-8 and IL-12^75,76^. Since immunomodulatory effects of BSp and Ash converge to separate spectrums of effector molecules, synergistic interaction between BSp and Ash may also be partially ascribed to their anti-inflammatory effect.

The present study demonstrated promising findings supporting the potential of combinatorial SFN-rich BSp and WA-rich Ash in inhibiting ER(−) BC. Since we have derived experimental findings utilizing transgenic mice, clinical benefits from this combinatorial treatment should be validated through further investigation on safety and efficacy of this combination. Though our study provides insights into possible molecular mechanisms underlying observed effects, further investigations are warranted to elucidate the molecular interplay between signaling pathways, transcription factors, and epigenetic regulations. While we reported shifting of the gut microbial composition due to combinatorial treatment, the implications of these alterations on cancer inhibition and relevant molecular mechanisms need to be addressed through further investigations.

## Methods

### Mouse model and animal housing

Transgenic mouse models C3(1)-SV40 Tag (FVB-Tg(C3-1-TAg) cJeg/JegJ) (C3) was used in this current investigation. The female mice of C3 mouse model typically develops tumors resembling Ductal Carcinoma in situ (DCIS) within the mammary epithelium at approximately 15 weeks of age due to transgene overexpression^22,23^. The breeder mice (~ 4 weeks of age) were obtained from Jackson Laboratory (Bar Harbor, ME). Experimental mice we genotyped (4 weeks of age) for transgene expression. Only transgene expressing female mice were used in this experiment. Mice were housed in the Animal Resource Facility of the University of Alabama at Birmingham (UAB, Birmingham, AL) and were maintained under the following conditions: 12-h dark/light cycle, 24 ± 2º C temperature with 40% to 60% humidity. Sample sizes were determined by a priori power calculation. A sample size of 10 mice/group was applied for detecting the effect of dietary interventions at a significance threshold of 0.05 with 80% power. The animal study was reviewed, approved, and performed in accordance with relevant guidelines and regulations by Institutional Animal Use and Care Committee of the University of Alabama at Birmingham (IACUC; Animal Project Numbers:10088 and 20653).

### Dietary treatment and experimental design

Standard AIN-93G diet was available commercially (TestDiet, St. Louis, MO, US). A customized BSp diet was formulated by supplementing AIN-93G with 26% SFN-rich BSp powder (Natural Sprout Co.) (Supplementary Table S3). Standard AIN-93G was supplemented with 0.16% human consumable Ashwagandha (Ash) extract (Shoden® Ashwagandha Extract, Nootropics Depot; ~ 6% WA) to prepare Ash diet (Supplementary Table S4). Combined BSp and Ash diets were prepared by adding 26% SFN-rich BSp powder and 0.16% Ash extract with AIN-93G diet (Supplementary Table S5). All the standard and customized diets were prepared by TestDiet. Transgene positive female C3 mice were randomly distributed into 4 groups (10–12 mice/group) at the age of 5 weeks. Different groups were treated with different dietary regimens starting from 6 weeks of age: (1) Control group: Mice were fed with control AIN93G diet; (2) BSp group: Mice were fed with BSp diet; (3) Ash group: Mice were fed with Ash diet; (4) Combined BSp and Ash diets: Mice were fed with Combined BSp and Ash diets. Detailed specification and nutrient composition of standard and customized diet are available in supplementary files.

### Growth performance and tumor observation

We recorded tumor volume and incidence of C3 mouse weekly. We also observed growth performance of experimental mice by monitoring body weight every other week. The formula below was used for calculating tumor volume: tumor volume (cm3) = (length × width2) × 0.523^72^. The experiment was terminated at 24 weeks of age when the mean tumor diameter in the control mice exceeded 1.0 cm. At the termination point, tumors tissue will be removed surgically, weighted, and stored in − 80 °C freezer for further investigation.

### Western blotting analysis

About 50 mg of frozen tumors tissue were subjected to total protein extraction with T-PER Tissue Protein Extraction Reagent (Thermo Fisher Scientific, MA, USA) following manufacturer’s protocol. Protein concentration was estimated by bicinchoninic acid assay (BCA) protein assay utilizing Pierce™ BCA Protein Assay Kits (Thermo Fisher Scientific, MA, USA). Protein was mixed with 4 × Laemmli Sample Buffer (Bio-Rad Laboratories, Inc., Hercules, CA, USA) and denatured at 95 °C for 5 min in the presence 2.5% βmercaptoethanol. An equal amount of protein was loaded on 4–15% NuPAGE Tris–HCl precast gels (Thermo Fisher Scientific, MA, USA) for protein separation. Separated proteins were transferred onto nitrocellulose membrane using Trans-Blot Turbo Transfer System (Bio-Rad, Hercules, CA, USA). The membranes were then blocked with 1% non-fat dry milk (Cell Signaling Technology, Inc., MA, USA) blocking buffer and probed with primary antibodies against p16, p21, p27, p53, p57, PTEN, BAX, BCL-2, PUMA, HDAC1, HDAC2, HDAC3, HDAC8, DNMT1, DNMT3A and DNMT3B (Cell Signaling Technology, Danvers, MA, USA). β-actin served as an internal control for each membrane. Following the incubation with secondary antibodies, immunoreactive bands were visualized using Clarity MaxTM Western ECL P Blotting Substrates (Bio-Rad, Hercules, CA, USA) using ChemiDocTM Imaging Systems (Bio-Rad, Hercules, CA, USA). Densitometric analysis of protein bands was performed with image analysis software Image J (v1.53e)^77^.

### RNA sequencing analysis

Total RNA was extracted from the tumor tissue of control diet and combined BSp and Ash diet treated mice as described previously^78^. Total RNA was used for RNA-seq pair-end library preparation (N_Control_ = 3, N_Combination_ = 3) and eventually sequenced on the Illumina Nextseq500 platform at the UAB Genomics Core Laboratories. Quality control and preprocessing of the raw data (FASTQ files) was performed with fastp tool for quality filtering (low quality, too many ns, etc.), length filtering (short reads), adapter trimming, and polyG tail trimming with the default parameter settings. Only clean and high quality fastq reads were aligned to the mouse reference genome GRCm39/mm39 using HISAT2^79^. The gene expression was qualified on aligned and sorted binary alignment and map files utilizing featureCounts. DeSeq2 was used for differential gene expression analysis^80^. The significant criterion of differentially expressed genes (DEGs) was set as |log2(FoldChange)|> 1 and false discovery rate (FDR) < 0.05. Other downstream analyses including principal component analysis (PCA), gene ontology (GO) enrichment analysis, and Kyoto Encyclopedia of Genes and Genomes (KEGG) pathway analysis were performed in R (v.4.1.1).

### Quantitative real-time PCR (RT-qPCR)

Validation of RNA sequencing analysis was performed with Quantitative real-time PCR for the target genes of interest including Hotairm1, Hdac9, Wnt6, Hoxa5, Sall1, Ntn4, β-actin. Equal amount of RNA (500 ng) was reverse-transcribed to cDNA using iScript™ cDNA Synthesis Kit (Bio-Rad, Hercules, CA, USA). PCR reactions were performed in triplicate with gene specific primers (Supplementary Table S6) purchased from Integrated DNA Technologies (Coralville, IA, USA) using SsoAdvanced Universal SYBR Green Supermix (Bio-Rad, Hercules, CA, USA). PCR amplification was performed in 20 μL wherein 1 μL of cDNA was used to amplify genes of interest with 500 nM reverse and forward primers and 1 × SYBR green master mix. The real-time PCR was performed using CFX Connect Real Time system (Bio-Rad, Hercules, CA, USA) with GAPDH (Glyceraldehyde 3-phospate dehydrogenase) as an endogenous control. Thermal cycling was initiated for 0.5 min at 95 °C followed by 45 cycles of PCR (94 °C for 15 s; 50 °C for 15 s, 60 °C for 30 s). Relative gene expression was calculated using 2− ΔΔCt method as described previously^81^.

### Gut microbiome analysis and 16S rRNA sequencing

Fecal samples (5–8 samples/group) were collected from control, BSp, WA, and combinatorial groups at two time points during the study: (1) Before tumor onset: 10 weeks of age. (2) After tumor onset: 20 weeks of age. Fecal DNA Isolation Kit, Zymo Research (Irvine, CA, USA) was used to extract genomic DNA according to the manufacturer’s instruction. The extracted DNA was quantified by microspectrophotometer (ThermoFisher, Waltham, MA, USA) and stored in fridge − 80 ºC for future analysis. An amplicon library was generated using PCR and barcoded primers from extracted DNA to amplify the 16S rRNA V4 region^82^. The forward and reverse primers (Eurofind Genomics, Inc., Huntsville, AL, USA) were as follows: Forward V4: 5′ AATGATACGGCGACCACCGAGATCTACACTATGGTAATTGTGTGCCAGCMGCCGCGGTAA-3′; Reverse V4: 5′CAAGAGAAGACGGCATACGAGATNNNNNNAGTCAGTCAGCCGGACTACHVGGGTWTCTAAT-3′. PCR products were quantified by PICO green dsDNA Reagent and purified by QIAquick gel Extraction Kit (Qiagen, Germantown, MD, USA) before the sequencing was performed using NextGen Sequencing Illumina MiSeq platform. FASTQ files were de-multiplexed and assessed for quality control using FastQC. Microbiome analysis was performed with the Quantitative Insight into Microbial Ecology (QIIME) data analysis package^83^. Samples were grouped into amplicon sequence variant (ASV) with 97% similarity by Uclust Clustering program that also was used to evaluate phylum level changes. PyNAST was used to generate multiple sequencing alignment^84^. Alpha diversity was analyzed with observed species, PD whole tree, Shannon and Simpson diversity indexes. Beta diversity (Bray–Curtis and weighed Unifrac) was calculated to quantify the dissimilarity between control and treatment groups. Statistical analysis of Bray–Curtis and weighed Unifrac tests of beta diversity was performed using permutational multivariate analysis of variance (PERMANOVA). Taxonomic abundance between groups were investigated using Kruskal Wallis test. Statistical significance after 5% false discovery rate (FDR) adjustment was presented.

### Statistical analyses

The sample size was estimated with a priori power calculation to obtain 95% power with a significance threshold of 0.05 utilizing GPower 3.1^85^. Statistical analysis was performed with GraphPad Prism version 10.0 (San Diego, CA, USA). Tumor incidence was analyzed with the Chi-Squared test. Results were represented as means ± standard error (SE) of the mean. The two-group comparisons were analyzed with unpaired t-test. The three or more groups’ comparisons were analyzed with one-way ANOVA and Tukey’s post-hoc test. Means that do not share a common superscript are significantly different at p ≤ 0.05. Combination index analysis with effect-based method (highest single agent model) and calculated with SiCoDEA^86^.

### Ethics approval

The animal study was reviewed, approved, and performed in accordance with relevant guidelines and regulations by Institutional Animal Use and Care Committee of the University of Alabama at Birmingham (IACUC; Animal Project Numbers:10088 and 20653). The study was also carried out in compliance with the Animal Research Reporting of In Vivo experiments (ARRIVE) guidelines.

## Conclusion

The current study, for the first time, gathers findings on the effect of BSp and Ash combinatorial treatment on any form of cancer in vivo. This investigation also provides compelling evidence that combined BSp and Ash administration may act synergistically against ER(−) BC both at the molecular and phenotypic level. Our results also indicated that combining BSp with Ash enhances targeting molecular components associated with multiple cell signaling pathway as well as epigenetic regulations. Furthermore, combined BSp and Ash intervention may confer health benefits through indirect influence on cellular processes by reshaping gut microbiota. Overall, BSp and Ash combination demonstrated substantial promise to be studied further for developing a novel strategy for inhibiting ER(−) BC through dietary intervention.

### Supplementary Information

Supplementary Information 1.

Supplementary Information 2.

## Data Availability

All data generated or analyzed during this study are included in this published article (and its Supplementary Information files).
