# Supplementary material for: A novel combinatorial approach using sulforaphane- and withaferin A-rich extracts for prevention of estrogen receptor-negative breast cancer through epigenetic and gut microbial mechanisms
Source: Sci Rep. 2024 May 27;14:12091. doi: 10.1038/s41598-024-62084-1 (PMC11130158; doi:10.1038/s41598-024-62084-1)
Supplement: Supplementary file 1 — Supplementary Information 1. [file 41598_2024_62084_MOESM1_ESM.pdf]

**Supplemental Table S1: Comparison of mammary tumor growth between different dietary treatments in C3 mice.**

| Dietary treatments | Inhibition rate (%) | Ratio of extended tumor latency (%) |
|---|---|---|
| Control | 0 | 0 |
| BSp | 27.5 | 13.3 |
| Ash | 29.4 | 16.5 |
| BSp+Ash | 75.1 | 21.5 |

***Inhibition rate on tumor growth*** *= (mean tumor weight at sacrifice of the control group – mean tumor weight at sacrifice of the treatment group)/mean tumor weight at sacrifice of the control group × 100%.*

***Ratio of extended tumor latency (%)*** *= (tumor latency in treatment group – tumor latency in control group)/tumor latency in control group × 100%.*

## Supplemental Figure S1

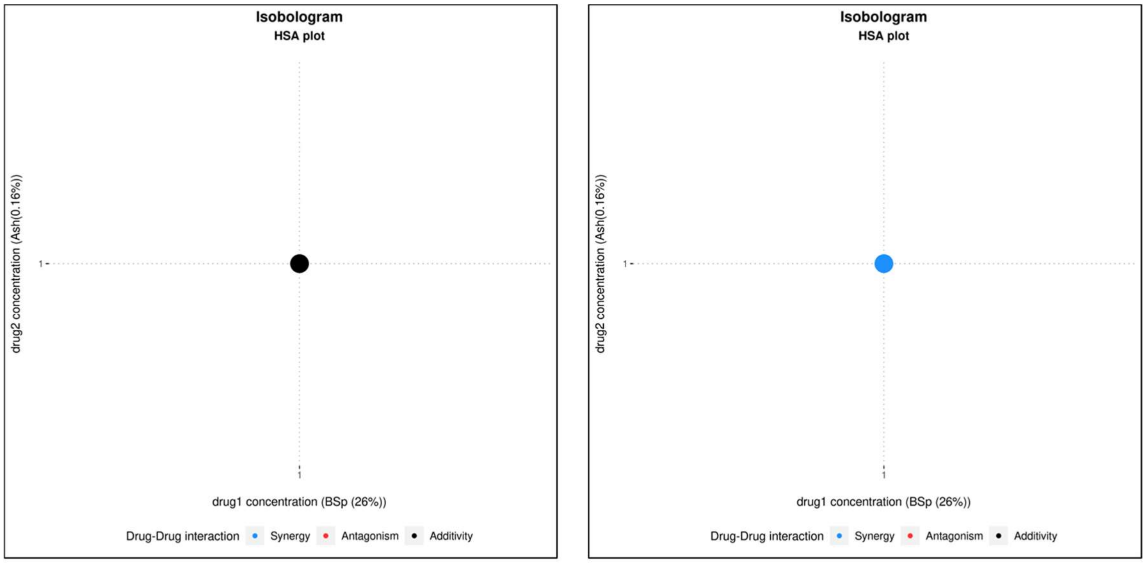


**Tumor Latency** **Tumor Weight**

**Supplemental Figure S1: Computation of Combination index (CI) with highest single agent (HAS) model using SiCoDEA.** The graph represents the isobologram generated based on the tumor latency (left panel) and tumor weight (right panel) results from BSp and Ash treatment alone and in combination in female C3 mice. The left isobologram represents an additive interaction between BSp and Ash for tumor latency and right isobologram indicates a synergistic interaction between BSp and Ash for tumor weight in C3 mice upon dietary treatment. Here, blue dots represent synergy, red dots represent antagonism, and black dots represent additivity. Dimensions of a dot are proportional to the strength of drug-drug interaction.

**Supplemental Figure S2**

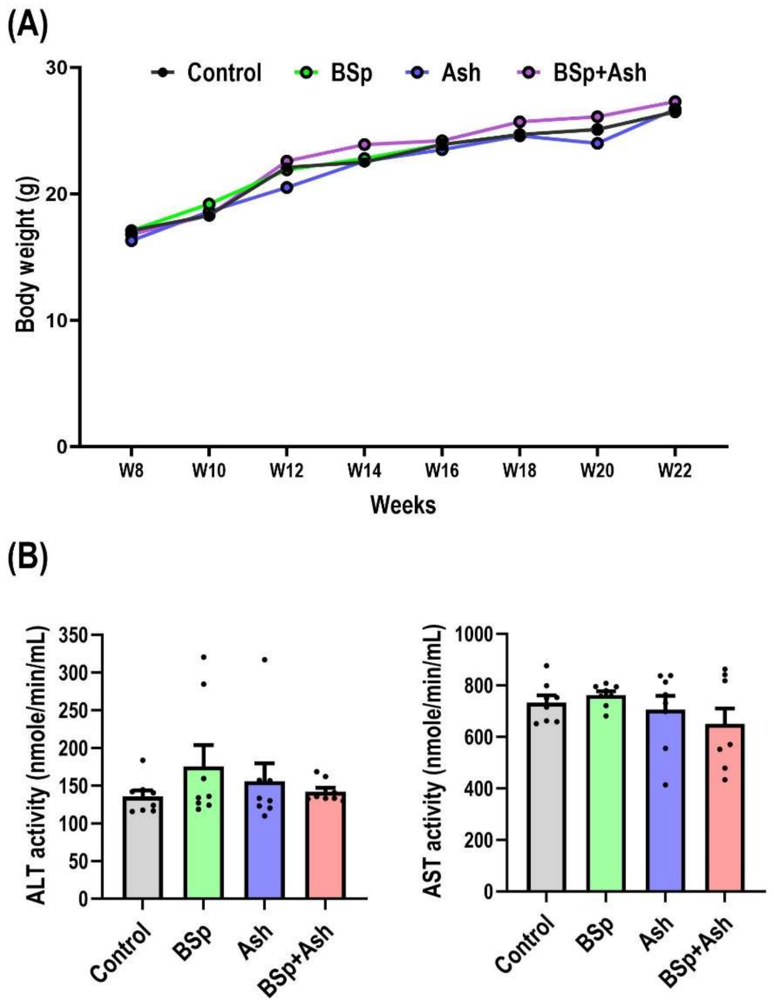


**Supplemental Figure S2**. **Effects of BSp and/or Ash supplementation on mouse growth performance or hepatic function in C3 mice.** The body weight (A) and hepatic function (B) of mouse treated with BSp and Ash diet alone and in combination. Values are means ± SEMs, n = 8-10. ALT and AST activity were measured to check the hepatic function of the mice treated with different diets. Comparisons of ALT and AST activity among the dietary groups were performed with One-way ANOVA analysis and Tukey's HSD. Here, BSp: Broccoli sprouts; Ash: Ashwagandha; BSp + Ash: Broccoli sprouts and Ashwagandha combination.

## Supplemental Figure S3

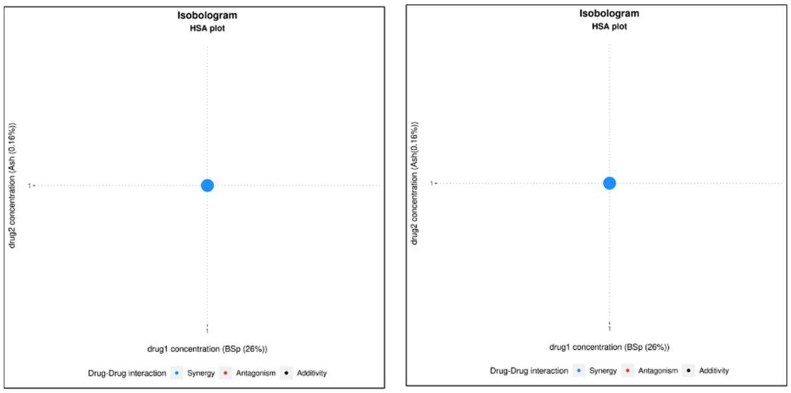


p53 p57

**Supplemental Figure S3: Computation of Combination index (CI) with highest single agent (HAS) model using SiCoDEA.** The graph represents the isobologram generated based on the cell cycle associated tumor suppressor p53 (left) and p57 (right) expression results from BSp and Ash treatment alone and in combination in female C3 mice. Here, blue dots represent synergy, red dots represent antagonism, and black dots represent additivity. Dimensions of a dot are proportional to the strength of drug-drug interaction.

## Supplemental Figure S4

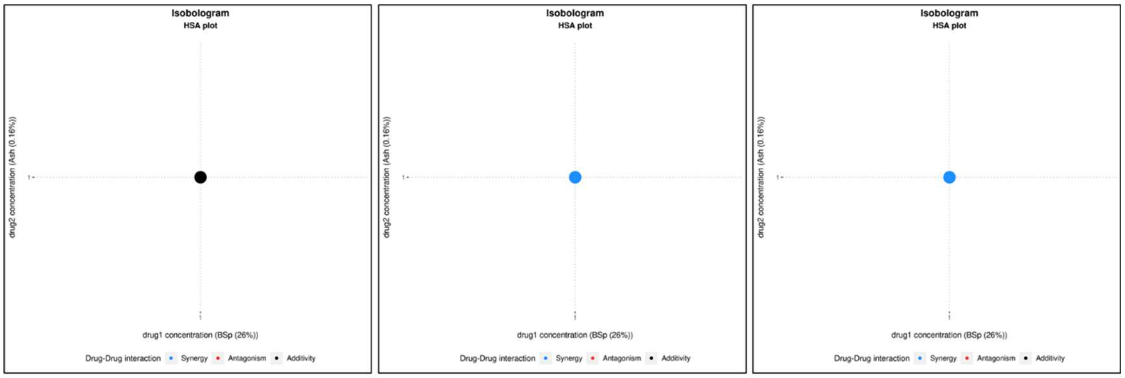


BAX PUMA BAX:BCL-2

**Supplemental Figure S5: Computation of Combination index (CI) with highest single agent (HAS) model using SiCoDEA.** The graph represents the isobologram generated based on the apoptosis associated protein BAX (left), PUMA (middle) and BAX: BCL-2 ratio (right) expression results from BSp and Ash treatment alone and in combination in female C3 mice. Here, blue dots represent synergy, red dots represent antagonism, and black dots represent additivity. Dimensions of a dot are proportional to the strength of drug-drug interaction.

## Supplemental Figure S5

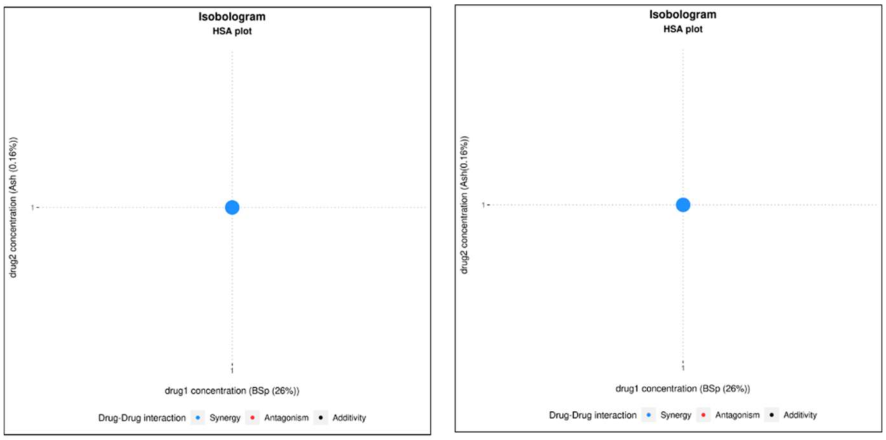


HDAC1 DNMT3A

**Supplemental Figure S5: Computation of Combination index (CI) with highest single agent (HAS) model using SiCoDEA.** The graph represents the isobologram generated based on the epigenetic machinery HDAC1 (left) and DNMT3A (right) expression results from BSp and Ash treatment alone and in combination in female C3 mice. Here, blue dots represent synergy, red dots represent antagonism, and black dots represent additivity. Dimensions of a dot are proportional to the strength of drug-drug interaction.

## Supplemental Figure S6

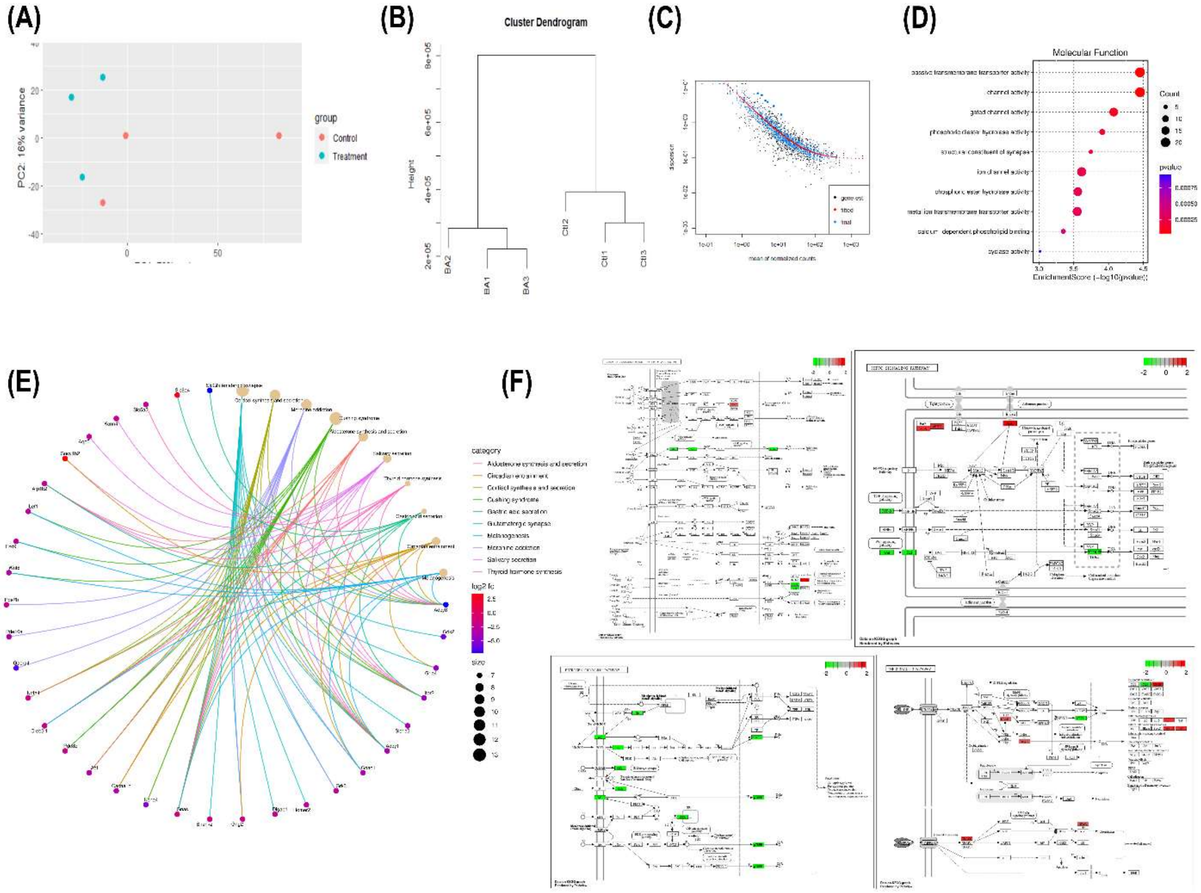


**Supplemental Figure S6**: **Dietary BSp and/or Ash Treatment Induce Alteration of Transcriptome in Mammary Tumor of C3 mice.** (A) Principal component analysis (PCA) plot shows the distribution of the control and treatment (combinatorial BSp + Ash group). We can see that the first two components (i.e., PC1 & PC2) separated the control (red dots) samples from treatment (blue dots) samples (B) Hierarchical clustering depicts the relationships among the samples. Here, Ctl indicates control diet treated control group samples while BA indicates combinatorial BSp + Ash treated test group samples. As shown, control samples and treatment samples are well separated in relation to each other. (C) Diagnostics plot for assessing the fit of the data to the DESeq2 model. A curve is fitted to the dispersion estimates for each gene. The data points scattered around the curve with decrease in dispersion as the mean expression level increases. (D) Dot plot shows top 10 enriched GO (Molecular Function) terms of DE mRNAs due to combinatorial treatment. Here, dot color is associated with the p-value. (E) The cnetplot for DEGs illustrating 10 enriched pathways in the KEGG analysis. Here, uniform-sized small dots symbolize DEGs (Differentially Expressed Genes), with purple indicating downregulation and red indicating upregulation. Enriched pathways are represented by yellow dots, and their size corresponds to the number of enriched genes. (E) Pathway graphs for DEG-relevant pathway visualization. Here, green shed indicates down regulation and res shed indicates upregulation.

**Supplemental Table S3: Detailed dietary ingredients and nutrient composition of the customized broccoli sprouts diet**

## Mod TestDiet® 57W5 w/26% Broccoli Sprout Seeds/Red — 5AJU

### DESCRIPTION

Modification of TestDiet® AIN-93G. 57W5, with 26% broccoli sprout seeds. Dyed red.

Intended for rodents in a laboratory setting.

CAUTION: Contains a new animal drug for investigational use only in laboratory research animals or for tests in vitro. Not for use in humans.

Storage conditions are particularly critical to TestDiet® products, due to the absence of antioxidants or preservative agents. To provide maximum protection against possible changes during storage, store in a dry, cool location. Storage under refrigeration (2° C) is recommended. Maximum shelf life is six months. (If long term studies are involved, storing the diet at -20° C or colder may prolong shelf life.) Be certain to keep in air tight containers.

| Product Forms Available* | Catalog # |
|---|---|
| 1/2" Pellet | 1814479 |

*Other Forms Available On Request

### INGREDIENTS (%)

| | |
|---|---|
| Broccoli Sprout Seeds | 26.0000 |
| Casein - Vitamin Tested | 20.0000 |
| Corn Starch | 13.6986 |
| Maltodextrin | 13.2000 |
| Sucrose | 10.0000 |
| Soybean Oil | 7.0000 |
| Powdered Cellulose | 5.0000 |
| AIN 93G Mineral Mix | 3.5000 |
| AIN 93 Vitamin Mix | 1.0000 |
| L-Cystine | 0.3000 |
| Choline Bitartrate | 0.2500 |
| FD&C Red No. 40 | 0.0500 |
| t-Butylhydroquinone | 0.0014 |

*See page 2 for Expanded Ingredient Listings

### FEEDING DIRECTIONS

Feed ad libitum. Plenty of fresh, clean water should be available at all times.

**CAUTION:**
**Perishable - store properly upon receipt.**
**For laboratory animal use only; NOT for human consumption.**

7/25/2022

### NUTRITIONAL PROFILE [1]

| | |
|---|---|
| ***Protein, %*** | ***25.6*** |
| Arginine, % | 0.70 |
| Histidine, % | 0.52 |
| Isoleucine, % | 0.96 |
| Leucine, % | 1.73 |
| Lysine, % | 1.45 |
| Methionine, % | 0.52 |
| Cystine, % | 0.37 |
| Phenylalanine, % | 0.96 |
| Tyrosine, % | 1.01 |
| Threonine, % | 0.77 |
| Tryptophan, % | 0.22 |
| Valine, % | 1.14 |
| Alanine, % | 0.55 |
| Aspartic Acid, % | 1.29 |
| Glutamic Acid, % | 4.08 |
| Glycine, % | 0.39 |
| Proline, % | 2.36 |
| Serine, % | 1.10 |
| Taurine, % | 0.00 |
| ***Fat, %*** | ***13.4*** |
| Cholesterol, ppm | 0 |
| Linoleic Acid, % | 3.58 |
| Linolenic Acid, % | 0.55 |
| Arachidonic Acid, % | 0.00 |
| Omega-3 Fatty Acids, % | 0.55 |
| Total Saturated Fatty A | 1.44 |
| Total Monounsaturated Fatty Acids, % | 2.33 |
| Polyunsaturated Fatty Acids, % | 8.94 |
| ***Fiber (max), %*** | ***11.4*** |
| ***Carbohydrates, %*** | ***47.2*** |
| ***Energy (kcal/g)*** [2] | ***4.12*** |

| From: | kcal | % |
|---|---|---|
| Protein | 1.024 | 24.9 |
| Fat (ether extract) | 1.204 | 29.3 |
| Carbohydrates | 1.887 | 45.9 |

| | |
|---|---|
| ***Minerals*** | |
| Calcium, % | 0.63 |
| Phosphorus, % | 0.32 |
| Potassium, % | 0.36 |
| Magnesium, % | 0.05 |
| Sodium, % | 0.18 |
| Chloride, % | 0.21 |
| Fluorine, ppm | 1.0 |
| Iron, ppm | 66 |
| Zinc, ppm | 35 |
| Manganese, ppm | 11 |
| Copper, ppm | 6.0 |
| Cobalt, ppm | 0.0 |
| Iodine, ppm | 0.21 |
| Chromium (added), ppm | 1.0 |
| Molybdenum, ppm | 0.14 |
| Selenium, ppm | 0.24 |
| ***Vitamins*** | |
| Vitamin A, IU/g | 5.3 |
| Vitamin D-3 (added), IU/g | 1.0 |
| Vitamin E, IU/kg | 81.6 |
| Vitamin K, ppm | 0.75 |
| Thiamin, ppm | 4.8 |
| Riboflavin, ppm | 6.7 |
| Niacin, ppm | 30 |
| Pantothenic Acid, ppm | 16 |
| Folic Acid, ppm | 2.1 |
| Pyridoxine, ppm | 5.8 |
| Biotin, ppm | 0.2 |
| Vitamin B-12, mcg/kg | 28 |
| Choline Chloride, ppm | 1,250 |
| Ascorbic Acid, ppm | 2.5 |

1. Formulation based on calculated values from the latest ingredient analysis information. Since nutrient composition of natural ingredients varies and some nutrient loss will occur due to manufacturing processes, analysis will differ accordingly. Nutrients expressed as percent of ration on an As-Fed basis except where otherwise indicated.
2. Energy (kcal/gm) - Sum of decimal fractions of protein, fat and carbohydrate x 4,9,4 kcal/gm respectively.

**NOTE: When assayed, actual levels may vary from calculated values.**

TestDiet

www.testdiet.com

**Supplemental Table S4: Detailed dietary ingredients and nutrient composition of the customized ashwagandha diet.**

## Mod TestDiet® w/ 0.16% Ashwagandha, Green — 5ZDW

### DESCRIPTION

Modification of TestDiet® AIN-93G, 57W5, with 0.16% ashwagandha. Dyed green.

Intended for rodents in a laboratory setting.

CAUTION: Contains a new animal drug for investigational use only in laboratory research animals or for tests in vitro. Not for use in humans.

Storage conditions are particularly critical to TestDiet® products, due to the absence of antioxidants or preservative agents. To provide maximum protection against possible changes during storage, store in a dry, cool location. Storage under refrigeration (2° C) is recommended. Maximum shelf life is six months. (If long term studies are involved, storing the diet at -20° C or colder may prolong shelf life.) Be certain to keep in air tight containers.

| ***Product Forms Available**** | ***Catalog #*** |
|---|---|
| 1/2" Pellet | 1819862-201 |

**Other Forms Available On Request*

### INGREDIENTS (%)

| | |
|---|---|
| Corn Starch | 39.5586 |
| Casein - Vitamin Tested | 20.0000 |
| Maltodextrin | 13.2000 |
| Sucrose | 10.0000 |
| Soybean Oil | 7.0000 |
| Powdered Cellulose | 5.0000 |
| AIN 93G Mineral Mix | 3.5000 |
| AIN 93 Vitamin Mix | 1.0000 |
| L-Cystine | 0.3000 |
| Choline Bitartrate | 0.2500 |
| Ashwagandha Extract | 0.1600 |
| Green (FD&C Blue No. 2, FD&C Blue No. 1 and FD&C Yellow No. 5) | 0.0300 |
| t-Butylhydroquinone | 0.0014 |

**See page 2 for Expanded Ingredient Listings*

### FEEDING DIRECTIONS

Feed ad libitum. Plenty of fresh, clean water should be available at all times.

**CAUTION:**
**Perishable - store properly upon receipt.**
**For laboratory animal use only; NOT for human consumption.**

7/26/2022

### NUTRITIONAL PROFILE [1]

| | |
|---|---|
| ***Protein, %*** | ***18.3*** |
| Arginine, % | 0.70 |
| Histidine, % | 0.52 |
| Isoleucine, % | 0.96 |
| Leucine, % | 1.73 |
| Lysine, % | 1.45 |
| Methionine, % | 0.52 |
| Cystine, % | 0.37 |
| Phenylalanine, % | 0.96 |
| Tyrosine, % | 1.01 |
| Threonine, % | 0.77 |
| Tryptophan, % | 0.22 |
| Valine, % | 1.14 |
| Alanine, % | 0.55 |
| Aspartic Acid, % | 1.29 |
| Glutamic Acid, % | 4.08 |
| Glycine, % | 0.39 |
| Proline, % | 2.36 |
| Serine, % | 1.10 |
| Taurine, % | 0.00 |
| ***Fat, %*** | ***7.1*** |
| Cholesterol, ppm | 0 |
| Linoleic Acid, % | 3.58 |
| Linolenic Acid, % | 0.55 |
| Arachidonic Acid, % | 0.00 |
| Omega-3 Fatty Acids, % | 0.55 |
| Total Saturated Fatty A | 1.05 |
| Total Monounsaturated Fatty Acids, % | 1.54 |
| Polyunsaturated Fatty Acids, % | 3.78 |
| ***Fiber (max), %*** | ***5.0*** |
| ***Carbohydrates, %*** | ***63.0*** |
| ***Energy (kcal/g)*** [2] | ***3.89*** |

| From: | kcal | % |
|---|---|---|
| Protein | 0.731 | 18.8 |
| Fat (ether extract) | 0.637 | 16.4 |
| Carbohydrates | 2.520 | 64.8 |

| ***Minerals*** | |
|---|---|
| Calcium, % | 0.51 |
| Phosphorus, % | 0.32 |
| Potassium, % | 0.36 |
| Magnesium, % | 0.05 |
| Sodium, % | 0.13 |
| Chloride, % | 0.22 |
| Fluorine, ppm | 1.0 |
| Iron, ppm | 40 |
| Zinc, ppm | 35 |
| Manganese, ppm | 11 |
| Copper, ppm | 6.0 |
| Cobalt, ppm | 0.0 |
| Iodine, ppm | 0.21 |
| Chromium (added), ppm | 1.0 |
| Molybdenum, ppm | 0.14 |
| Selenium, ppm | 0.24 |

| ***Vitamins*** | |
|---|---|
| Vitamin A, IU/g | 4.0 |
| Vitamin D-3 (added), IU/g | 1.0 |
| Vitamin E, IU/kg | 81.6 |
| Vitamin K, ppm | 0.75 |
| Thiamin, ppm | 4.8 |
| Riboflavin, ppm | 6.7 |
| Niacin, ppm | 30 |
| Pantothenic Acid, ppm | 16 |
| Folic Acid, ppm | 2.1 |
| Pyridoxine, ppm | 5.8 |
| Biotin, ppm | 0.2 |
| Vitamin B-12, mcg/kg | 28 |
| Choline Chloride, ppm | 1,250 |
| Ascorbic Acid, ppm | 0.0 |

1. Formulation based on calculated values from the latest ingredient analysis information. Since nutrient composition of natural ingredients varies and some nutrient loss will occur due to manufacturing processes, analysis will differ accordingly. Nutrients expressed as percent of ration on an As-Fed basis except where otherwise indicated.
2. Energy (kcal/gm) - Sum of decimal fractions of protein, fat and carbohydrate x 4,9,4 kcal/gm respectively.

**NOTE: When assayed, actual levels may vary from calculated values.**

TestDiet
www.testdiet.com

**Supplemental Table S5: Detailed dietary ingredients and nutrient composition of the customized broccoli sprouts and ashwagandha diet.**

## Mod TestDiet® 57W5 w/ 26% Broccoli Sprouts and 0.16% Ashw 5ZDX

### DESCRIPTION

Modification of TestDiet® AIN-93G, 57W5, with 26% broccoli sprout seeds and 0.16% ashwagandha. Dyed yellow.

Intended for rodents in a laboratory setting.

CAUTION: Contains a new animal drug for investigational use only in laboratory research animals or for tests in vitro. Not for use in humans.

Storage conditions are particularly critical to TestDiet® products, due to the absence of antioxidants or preservative agents. To provide maximum protection against possible changes during storage, store in a dry, cool location. Storage under refrigeration (2° C) is recommended. Maximum shelf life is six months. (If long term studies are involved, storing the diet at -20° C or colder may prolong shelf life.) Be certain to keep in air tight containers.

| *Product Forms Available** | *Catalog #* |
|---|---|
| 1/2" Pellet | 1819863-201 |

*Other Forms Available On Request

### INGREDIENTS (%)

| | |
|---|---|
| Broccoli Sprout Seeds | 26.0000 |
| Casein - Vitamin Tested | 20.0000 |
| Corn Starch | 13.5586 |
| Maltodextrin | 13.2000 |
| Sucrose | 10.0000 |
| Soybean Oil | 7.0000 |
| Powdered Cellulose | 5.0000 |
| AIN 93G Mineral Mix | 3.5000 |
| AIN 93 Vitamin Mix | 1.0000 |
| L-Cystine | 0.3000 |
| Choline Bitartrate | 0.2500 |
| Ashwagandha Extract | 0.1600 |
| FD&C Yellow No. 5 | 0.0300 |
| t-Butylhydroquinone | 0.0014 |

*See page 2 for Expanded Ingredient Listings*

### FEEDING DIRECTIONS

Feed ad libitum. Plenty of fresh, clean water should be available at all times.

**CAUTION:**
**Perishable - store properly upon receipt.**
**For laboratory animal use only; NOT for human consumption.**

7/26/2022

### NUTRITIONAL PROFILE [1]

| | |
|---|---|
| ***Protein, %*** | ***25.6*** |
| Arginine, % | 0.70 |
| Histidine, % | 0.52 |
| Isoleucine, % | 0.96 |
| Leucine, % | 1.73 |
| Lysine, % | 1.45 |
| Methionine, % | 0.52 |
| Cystine, % | 0.37 |
| Phenylalanine, % | 0.96 |
| Tyrosine, % | 1.01 |
| Threonine, % | 0.77 |
| Tryptophan, % | 0.22 |
| Valine, % | 1.14 |
| Alanine, % | 0.55 |
| Aspartic Acid, % | 1.29 |
| Glutamic Acid, % | 4.08 |
| Glycine, % | 0.39 |
| Proline, % | 2.36 |
| Serine, % | 1.10 |
| Taurine, % | 0.00 |
| ***Fat, %*** | ***13.4*** |
| Cholesterol, ppm | 0 |
| Linoleic Acid, % | 3.58 |
| Linolenic Acid, % | 0.55 |
| Arachidonic Acid, % | 0.00 |
| Omega-3 Fatty Acids, % | 0.55 |
| Total Saturated Fatty A | 1.44 |
| Total Monounsaturated Fatty Acids, % | 2.33 |
| Polyunsaturated Fatty Acids, % | 8.94 |
| ***Fiber (max), %*** | ***11.4*** |
| ***Carbohydrates, %*** | ***47.0*** |
| ***Energy (kcal/g)*** [2] | ***4.11*** |

| From: | kcal | % |
|---|---|---|
| Protein | 1.024 | 24.9 |
| Fat (ether extract) | 1.204 | 29.3 |
| Carbohydrates | 1.882 | 45.8 |

| ***Minerals*** | |
|---|---|
| Calcium, % | 0.63 |
| Phosphorus, % | 0.32 |
| Potassium, % | 0.36 |
| Magnesium, % | 0.05 |
| Sodium, % | 0.18 |
| Chloride, % | 0.21 |
| Fluorine, ppm | 1.0 |
| Iron, ppm | 66 |
| Zinc, ppm | 35 |
| Manganese, ppm | 11 |
| Copper, ppm | 6.0 |
| Cobalt, ppm | 0.0 |
| Iodine, ppm | 0.21 |
| Chromium (added), ppm | 1.0 |
| Molybdenum, ppm | 0.14 |
| Selenium, ppm | 0.24 |
| ***Vitamins*** | |
| Vitamin A, IU/g | 5.3 |
| Vitamin D-3 (added), IU/g | 1.0 |
| Vitamin E, IU/kg | 81.6 |
| Vitamin K, ppm | 0.75 |
| Thiamin, ppm | 4.8 |
| Riboflavin, ppm | 6.7 |
| Niacin, ppm | 30 |
| Pantothenic Acid, ppm | 16 |
| Folic Acid, ppm | 2.1 |
| Pyridoxine, ppm | 5.8 |
| Biotin, ppm | 0.2 |
| Vitamin B-12, mcg/kg | 28 |
| Choline Chloride, ppm | 1,250 |
| Ascorbic Acid, ppm | 2.5 |

1. Formulation based on calculated values from the latest ingredient analysis information. Since nutrient composition of natural ingredients varies and some nutrient loss will occur due to manufacturing processes, analysis will differ accordingly. Nutrients expressed as percent of ration on an As-Fed basis except where otherwise indicated.
2. Energy (kcal/gm) - Sum of decimal fractions of protein, fat and carbohydrate x 4,9,4 kcal/gm respectively.

**NOTE: When assayed, actual levels may vary from calculated values.**

TestDiet
www.testdiet.com

## Supplemental Table S6: List of primers used for quantitative PCR*.

| Gene | Forward Primer (FP) | Forward Primer (FP) |
|---|---|---|
| Hotairm1 | AGCTGGGAGATTAATCAACC | GAGTTCCCTTACCAAGCTGC |
| Hdac9 | CAGAAGCAGCACGAGAATTTGA | CTCTCTGCGATGCCTCTCTAC |
| Wnt6 | CTCCTACAGTGTGGTTGTCAGG | GCGCATCCATAAAGAGTCTTGA |
| Hoxa5 | CTCATTTTGCGGTCGCTATCC | ATCCATGCCATTGTAGCCGTA |
| Shc3 | GCCGGGAGTCACCTATGTG | TTCCCTGGTAACCTGAGTTCT |
| Sall1 | CTCAACATTTCCAATCCGACCC | GGCATCCTTGCTCTTAGTGGG |
| Ntn4 | GCAGGCTTGAATGGAGTAGC | GCAGCGTTGCATTTATCACAC |
| β-actin | GGCTGTATTCCCCTCCATCG | CCAGTTGGTAACAATGCCATGT |

'*' all sequences are 5' to 3'.

## Supplemental Figure S7

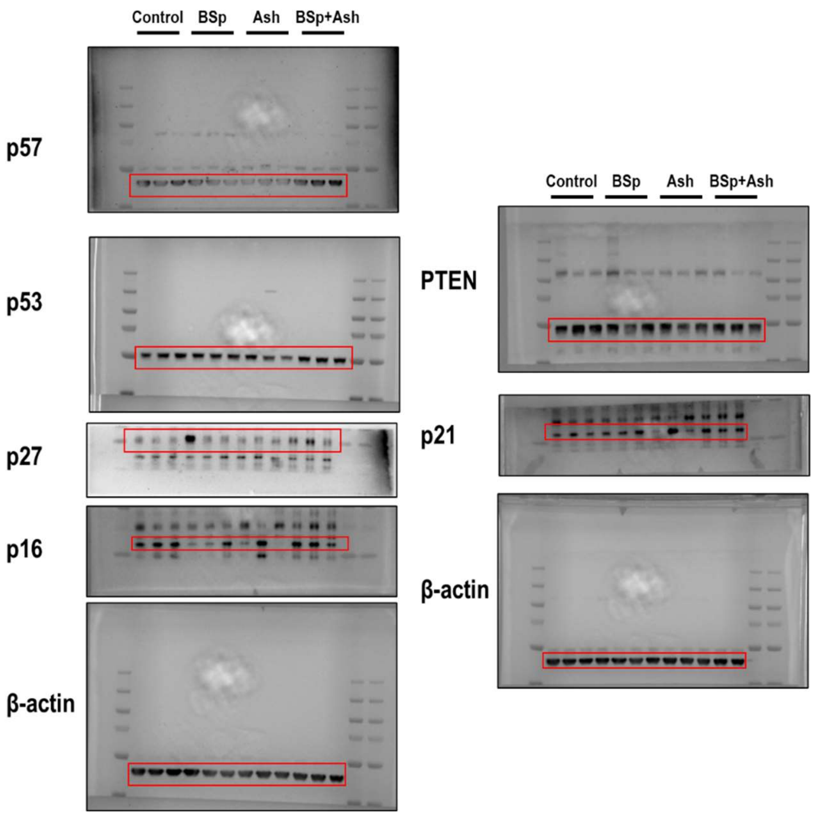


**Original Blot Images for Figure 2 (2A)**

Supplemental Figure S8

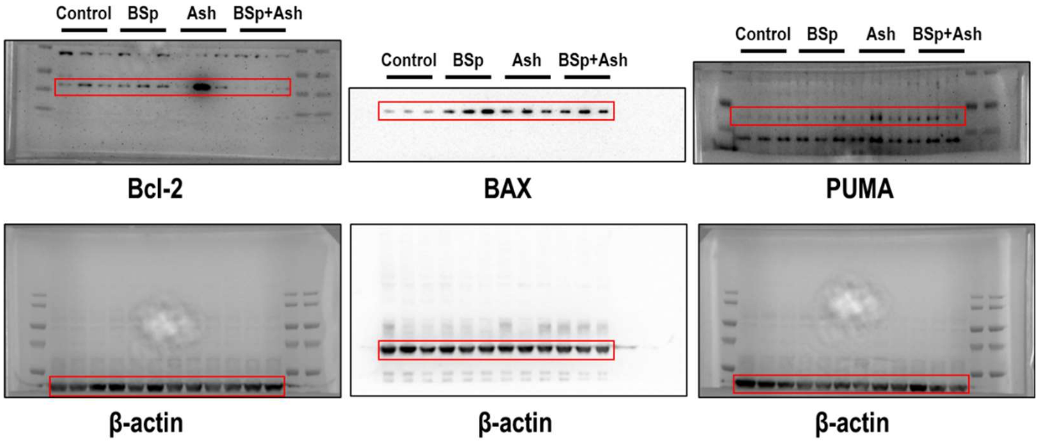


Original Blot Images for Figure 3 (3A)

Supplemental Figure S9

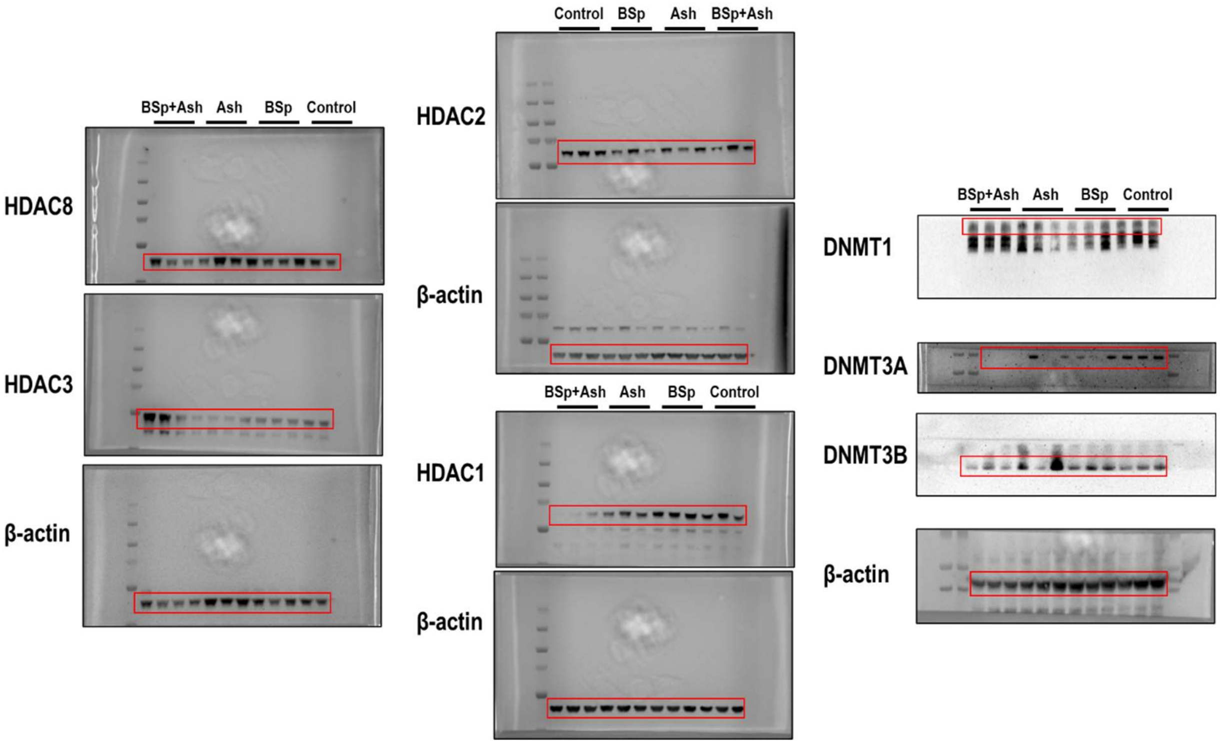


Original Blot Images for Figure 4 (4A)
